# HIV-1 neutralizing antibodies elicited in humans by a prefusion-stabilized envelope trimer form a reproducible class targeting fusion peptide

**DOI:** 10.1016/j.celrep.2023.112755

**Published:** 2023-07-11

**Authors:** Shuishu Wang, Flavio Matassoli, Baoshan Zhang, Tracy Liu, Chen-Hsiang Shen, Tatsiana Bylund, Timothy Johnston, Amy R. Henry, I-Ting Teng, Prabhanshu Tripathi, Jordan E. Becker, Anita Changela, Ridhi Chaudhary, Cheng Cheng, Martin Gaudinski, Jason Gorman, Darcy R. Harris, Myungjin Lee, Nicholas C. Morano, Laura Novik, Sijy O’Dell, Adam S. Olia, Danealle K. Parchment, Reda Rawi, Jesmine Roberts-Torres, Tyler Stephens, Yaroslav Tsybovsky, Danyi Wang, David J. Van Wazer, Tongqing Zhou, Nicole A. Doria-Rose, Richard A. Koup, Lawrence Shapiro, Daniel C. Douek, Adrian B. McDermott, Peter D. Kwong

**Affiliations:** 1 Vaccine Research Center, National Institutes of Health, Bethesda, MD 20892, USA; 2 Zuckerman Mind Brain Behavior Institute, Columbia University, New York, NY 10027, USA; 3 Department of Biochemistry and Molecular Biophysics, Columbia University Vagelos College of Physicians and Surgeons, New York, NY 10032, USA; 4 Electron Microscopy Laboratory, Cancer Research Technology Program, Frederick National Laboratory for Cancer Research, Frederick, MD 21701, USA; 5 Lead contact

## Abstract

Elicitation of antibodies that neutralize the tier-2 neutralization-resistant isolates that typify HIV-1 transmission has been a long-sought goal. Success with prefusion-stabilized envelope trimers eliciting autologous neutralizing antibodies has been reported in multiple vaccine-test species, though not in humans. To investigate elicitation of HIV-1 neutralizing antibodies in humans, here, we analyze B cells from a phase I clinical trial of the “DS-SOSIP”-stabilized envelope trimer from strain BG505, identifying two antibodies, N751-2C06.01 and N751-2C09.01 (named for donor-lineage.clone), that neutralize the autologous tier-2 strain, BG505. Though derived from distinct lineages, these antibodies form a reproducible antibody class that targets the HIV-1 fusion peptide. Both antibodies are highly strain specific, which we attribute to their partial recognition of a BG505-specific glycan hole and to their binding requirements for a few BG505-specific residues. Prefusion-stabilized envelope trimers can thus elicit autologous tier-2 neutralizing antibodies in humans, with initially identified neutralizing antibodies recognizing the fusion-peptide site of vulnerability.

## In brief

Vaccine elicitation of tier-2 HIV-1 neutralizing antibodies in humans has been a long-sought goal. Wang et al. isolate and determine structures of two antibodies from a clinical trial of a prefusion-closed HIV-1 envelope trimer. These antibodies target the fusion-peptide site of vulnerability, form a reproducible class, and neutralize the tier-2 HIV-1 strain BG505.

## Graphical Abstract

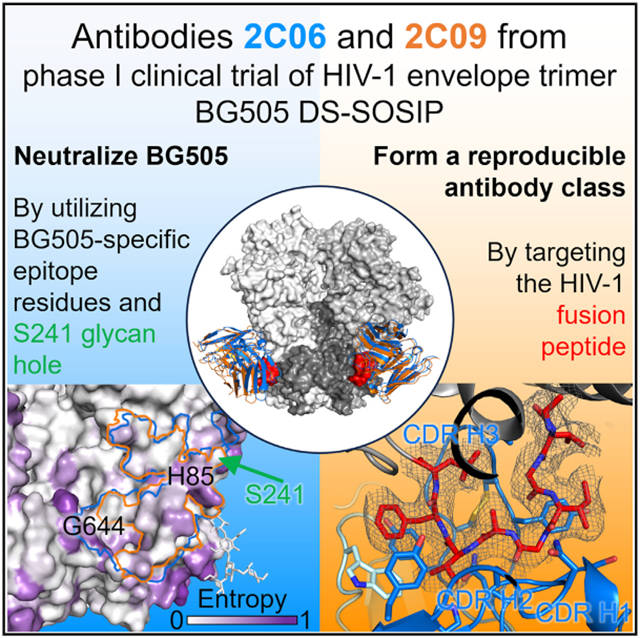

## INTRODUCTION

The HIV-1 envelope (Env) trimer is protected by multiple mechanisms of humoral evasion, including conformational masking, glycan shielding, and sequence variation.^1-3^ While HIV-1 infection generally elicits autologous neutralizing antibodies^4,5^—and in a small fraction of individuals even broadly neutralizing antibodies^6-12^—Env-based immunization has generally yielded antibodies that are incapable of neutralizing the tier-2 neutraliza-tion-resistant strains of HIV-1 that typify natural transmission. A major roadblock has been trimer instability, with the prefusion mature Env trimer changing conformations and ultimately disassembling into constituent gp120 and gp41 subunits and in the process exposing immunodominant surfaces, which elicit poorly or non-neutralizing antibodies.

Stabilization of the Env trimer through the incorporation of a disulfide (SOS) between residues 501 of gp120 and 605 of gp41 and the substitution of an isoleucine to a proline (IP) helped fix the trimer into a prefusion-closed conformation recognized by broadly neutralizing antibodies but not by non-neutralizing antibodies.^13^ SOSIP-stabilized Env trimers could elicit autologous neutralizing antibodies in rabbits against diverse HIV strains,^14^ including the clade A strain BG505 and the clade B strains B41 and AMC008. These often target glycan holes, such as at residues 241 and 289 in strain BG505.^15,16^

While vaccine elicitation of tier-2 neutralizing responses has now been reported in multiple vaccine-test species including non-human primates,^17-19^ the characteristics of neutralizing antibodies elicited in humans by Env stabilized in a prefusion-closed conformation have not been reported. How does the human immune system respond to conformationally stabilized and highly glycosylated immunogens? Here, we analyze B cells from a phase I clinical trial (VRC 018) assessing a prefusion-stabilized Env trimer from strain BG505, fixed with an additional disulfide (201C–433C) to comprise DS-SOSIP, which stays in a prefusion-closed conformation even in the presence of human CD4.^20,21^ We sorted B cells with stabilized trimers with their bases covered with glycans and sequenced and expressed constituent antibodies, for which we assessed binding and neutralization. For antibodies capable of neutralizing the autologous strain, BG505, we determined cryo-electron microscopy (cryo-EM) structures with Env trimer. Collectively, the results reveal only a minority of responding B cells to be directed to the glycan-dense surface of the Env trimer versus its glycan-free base, and of these B cells, only ~1 in 100 bound with sufficient affinity to neutralize autologous virus; overall, we report Env-trimer vaccine-elicited antibodies from humans capable of neutralizing a tier-2 neutralization-resistant isolate to be strain-specific members of a reproducible class of antibodies, targeting the fusion-peptide site of vulnerability.

## RESULTS

### Sorting of VRC 018 B cells from donor N751 reveal that only a small fraction of elicited antibodies recognize the glycan-dense surface of the Env trimer

**Figure 1.**
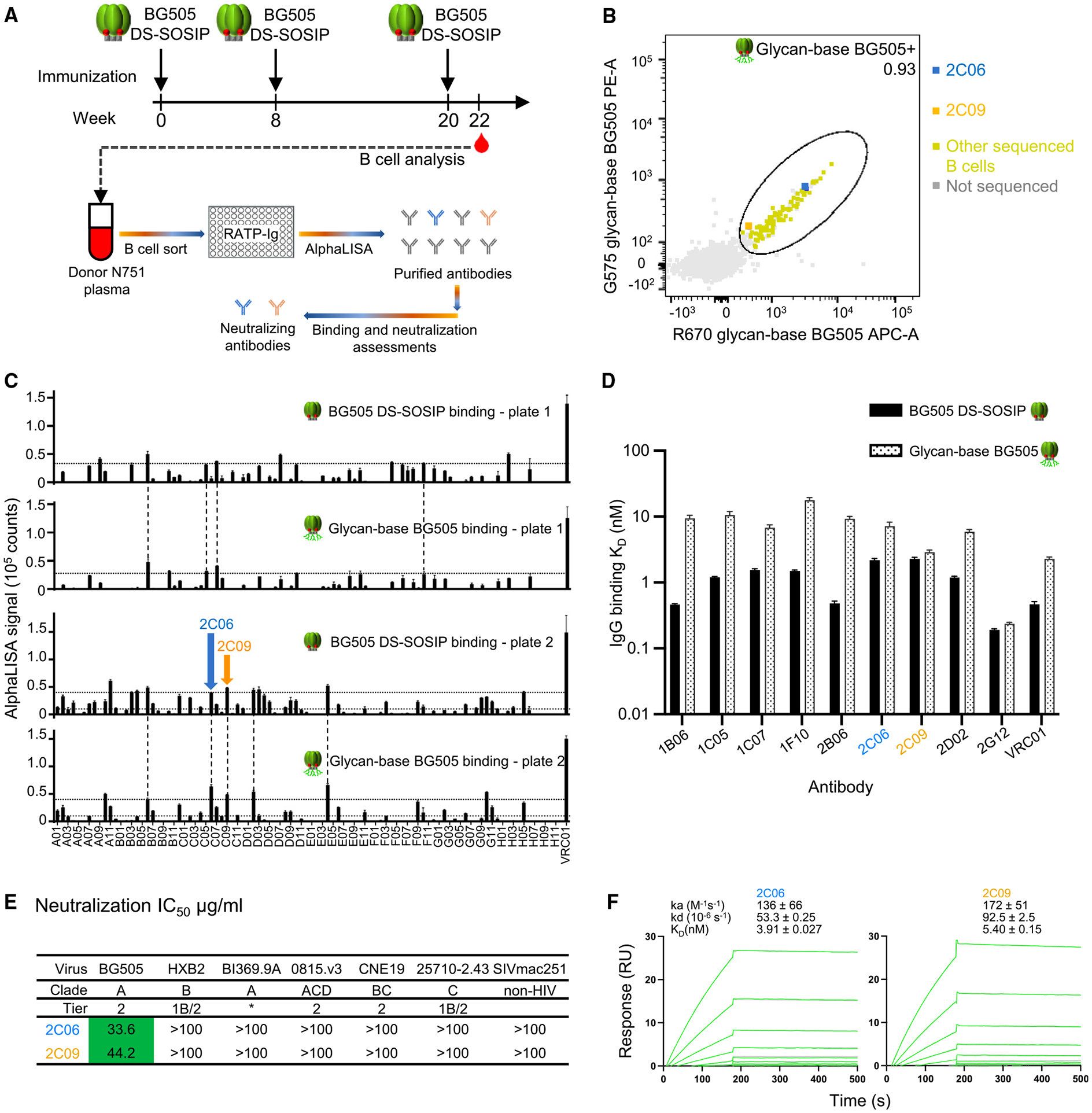
Sorting of VRC 018 B cells from donor N751 identifies antibodies 2C06 and 2C09 that neutralize BG505 (A) VRC 018 clinical regimen and a flowchart for B cell sorting and antibody screening. (B) Single B cell sorting with glycan-base BG505 trimer probes reveals only a small fraction of memory B cells from VRC 018 clinical trial to be directed to the glycan-dense surface of the Env trimer versus its glycan-free base. (C) AlphaLISA screening of RATP-Ig supernatants from sorted B cells for antibodies that bind both BG505 DS-SOSIP and glycan-base BG505. Data were measured in triplicates; error bars represent standard error of the mean (SEM). (D) Apparent affinity measurement of top antibodies from AlphaLISA screening for binding to BG505 DS-SOSIP and glycan-base BG505 trimers. Antibody IgGs were expressed and purified, and their binding to trimers was measured by Carterra. 2G12 and VRC01 were used as positive controls. Motavizumab was used as negative control, and no binding was observed. (E) Antibodies 2C06 and 2C09 neutralize BG505, but no other tested strains. Asterisk denotes tier status for BI369.9A unknown but resistant to antibodies 17b, 48d, F105, 3074, and 447-52D that neutralize only laboratory-adapted strains.^24^ Neutralization for other antibodies from (D) that bind glycan-base BG505 are shown in Figure S2. (F) Affinity of antibody Fabs binding to BG505 DS-SOSIP trimer, measured by SPR. Data in (D) and (F) were measured once and the curves fitted with a simple 1:1 Langmuir binding model for the reported mean ± SEM. See also Figures S1 and S2.

In the Vaccine Research Center (VRC) 018 clinical trial (ClinicalTrials.gov NCT03783130),^22^ although no neutralization activity against the autologous BG505 strain was detected in serum samples, BG505 DS-SOSIP-specific antibody responses were elicited in all groups vaccinated with three 500-μg doses of BG505 DS-SOSIP adjuvanted with alum at 2 weeks after regimen completion. This suggested that neutralizing antibodies might have been elicited, but at titers too low to be detected in bulk sera. To characterize vaccine-elicited antibodies, we sorted B cells from donor N751, which showed the highest BG505 DS-SOSIP-reactive ELISA responses in the 500-μg intramuscular group of five participants^22^ (Figures 1A, 1B, and S1). For sorting, we used a modified BG505 Env trimer with additional stabilization mutations^23^ along with two engineered *N*-linked glycans per protomer, which were designed to cover the trimer base (hereafter referred to as glycan-base BG505 trimer, Figures S1A and S1B) to reduce the background of dominant base-targeting antibodies, allowing for the selection of antibodies binding to neutralizing epitopes on the Env trimer.

To quantify the number of cells binding to the glycan-dense Env-trimer surface relative to those bound the glycan-free base, we performed cell cytometry on immunoglobulin G-positive (IgG^+^) memory B cells (singlet, live, CD19^+^, CD3^−^, CD14^−^, CD56^−^, IgG^+^) from donor N751 (Figures S1C and S1D). Of antigen-positive memory B cells, 12.9% bound the glycan-base-covered BG505 versus 87.1% which bound only to BG505 with protein base. These data suggest that about 1 in 10 antigen-positive B cells could recognize the glycan-dense surface of the Env trimer.

From two plates of single-sorted B cells, 96 wells each, specific for the glycan-base BG505 trimer, we sequenced immunoglobulin genes and cloned them into an expression vector utilizing a method for rapid assembly, transfection, and production of immunoglobulins (RATP-Ig).^25^ RATP-Ig supernatants were screened for binding to BG505 DS-SOSIP and glycan-base BG505 Env trimers by AlphaLISA, and we identified nine double-positive B cell supernatants (Figure 1C). One of clones did not express in transient transfection of mammalian cells; the other eight were purified and confirmed by surface plasmon resonance (SPR) to bind glycan-base BG505 (dissociation constant [K_D_] values ranging from 2.86 to 17.6 nM) and BG505 DS-SOSIP (K_D_ values ranging from 0.46 to 2.28 nM) (Figure 1D). Overall, only a small fraction of the antigen-positive memory B cells encoded antibodies with moderate binding affinity to the non-base glycan-dense surface of the Env-trimer immunogen.

### Vaccine-elicited human antibodies N751-2C06.01 and N751-2C09.01 neutralize HIV strain BG505

We further characterized the purified antibodies for neutralization activity and found that the antibodies N751-2C06.01 and N751-2C09.01 (named for donor-lineage.clone, hereafter referred to as 2C06 and 2C09) neutralized both BG505 and a version of BG505 virus with glycan at residue 611 missing (BG505.N611Q), found to be especially sensitive to fusion-peptide-directed antibodies (Figures 1E, S2A, and S2B). Because of their ability to neutralize the BG505.N611Q virus, we tested antibodies 2C06 and 2C09 for their binding to the exposed N-terminal segment of the fusion peptide (Figure S2C). Both antibodies bound fusion peptide, and 2C06 had substantially reduced affinity to shorter peptides; 2C09 also showed reduced binding to shorter peptides, though not as significantly as 2C06. Both 2C06 and 2C09 competed with fusion-peptide-directed antibodies VRC34.01, PGT151, and vFP16.02 for binding to BG505 Env trimer (Figure S2D).

We used SPR to measure the binding of the antigen-binding fragments (Fabs) of 2C06 and 2C09 to BG505 DS-SOSIP trimer. Both bound tightly, with K_D_ of 3.91 and 5.40 nM for 2C06 and 2C09, respectively (Figure 1F). Overall, we identified two antibodies from the BG505 DS-SOSIP/alum clinical trial that neutralized wild-type BG505, appeared to be fusion-peptide directed, and bound BG505 Env trimer with nanomolar affinity.

### Cryo-EM structure of N751-2C06.01 in complex with BG505 DS-SOSIP reveals the antibody to bind at the fusion-peptide site of vulnerability

To define recognition details, we determined the cryo-EM structure of 2C06 in complex with BG505 DS-SOSIP. We obtained a 3D-reconstruction map at 2.95 Å from 645,442 particles utilizing C1 symmetry (Figure S3 and Table S1). Binding of 2C06 induced asymmetry in the trimer (Figure S3F); specifically, interactions of complementarity-determining region 3 of heavy chain (CDR H3) from two of the Fabs with the C-terminal helix of a neighboring gp41 subunit served to position this helix closer to the two Fabs and to the primary protomer. This movement of gp41 C-terminal helix was observed with two of the Fabs, not the third, and enabled the two N-terminal residues from the third fusion peptide (residues 512 and 513) to interact with the C-terminal helix of a neighboring gp41.

**Figure 2.**
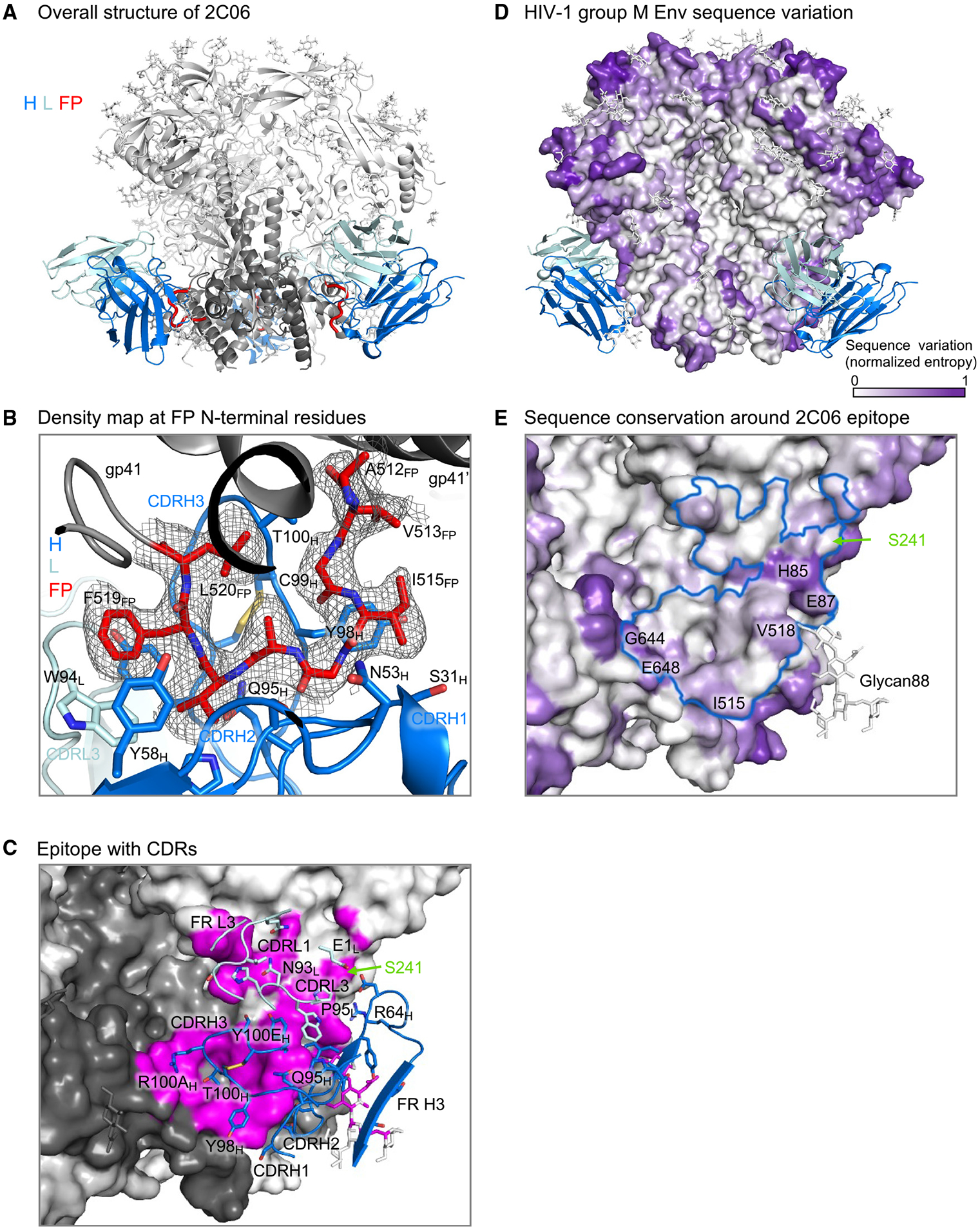
Cryo-EM structure of N751-2C06.01 in complex with BG505 DS-SOSIP reveals antibody recognition at the fusion-peptide site of vulnerability (A) Cartoon representation of 2C06 in complex with BG505 DS-SOSIP. 2C06 bound at the fusion-peptide site and interacted with fusion peptide (FP), shown in red. Antibody heavy chain (H) is shown in blue, light chain (L) in pale cyan, gp41 in dark gray, and gp120 in light gray. (B) Density map for fusion peptide N-terminal residues. The structure is shown in cartoon representation, with fusion peptide and antibody side chains interacting with fusion peptide shown in sticks. Color codes are as in (A). (C) 2C06 epitope details. Env trimer is shown as surface with subunits in different shades of gray and the epitope surface in magenta. Antibody CDRs and FRs involved in binding are shown in cartoon representation, with interacting side chains shown in sticks. The glycan hole at S241 is on the edge of the epitope and is labeled. (D) Overall sequence variation of HIV-1 group M Env glycoproteins. Normalized sequence entropy is mapped on the trimer surface, with absolutely conserved residues (entropy of 0) in white and the most diverse residues (entropy of 1) in purple. (E) HIV-1 Env sequence variation around the 2C06 epitope. 2C06 epitope is shown as a blue outline. Part of the 2C06 epitope is highly variable (labeled). The residue at 241 is well conserved with over 96% of isolates having an *N*-linked glycan sequon, whereas BG505 has a glycan hole. See also Figure S3 and Tables S1-S3.

Antibody 2C06 bound at the fusion-peptide site of vulnerability (Figure 2A), with epitope analysis utilizing prior antibody-Env structural complexes^26^ indicating substantial epitope overlap with VRC34.01 (71%) and lower epitope overlap with ACS202 (62%) and PGT151 (54%) (Table S2). Fusion-peptide residues 514–520 interacted almost exclusively with the heavy chain of 2C06 (Figure 2B); these residues formed a U shape, looping back to interact with trimer. As mentioned above, at the binding sites where the 2C06 CDR H3 had less interaction with the neighboring gp41, the N-terminal two residues of the fusion peptide interacted with the C-terminal gp41 helix and were resolved with relatively weak electron density, whereas in the other two gp41 subunits the first two N-terminal residues of the fusion peptide did not have discernible electron density.

The fusion peptide was flanked by CDRs H1 and H2 on one side and CDR H3 on the other (Figure 2B), and its binding involved an average of ~470 Å^2^ of buried surface area (BSA) (Tables S3 and S4). CDR L3 had minor interactions with fusion-peptide residues 518 and 519 at ~60 Å^2^ BSA, and interactions with CDRs of heavy and light chains together accounted for nearly half of the total protein BSA of ~1,200 Å^2^, of which more than 800 Å^2^ were from the heavy chain. Light-chain interactions involved CDRs L1 and L3, framework region 3 (FR L3), and the side chain of its N-terminal residue (Figure 2C). Most light-chain interactions were with residues leading up to glycan at N88 (glycan88), which shared interactions with heavy chain, with glycan88 covering ~250 Å^2^ of heavy-chain BSA (Tables S3 and S4).

The 2C06 epitope also included S241, which is an *N*-glycosylation site in 97% of HIV isolates (http://www.hiv.lanl.gov/content/index), with BG505 being one of the rare strains with a glycan hole at 241 (Figure 2C and Table S5). HIV-1 group M Env surface residues are highly variable, especially at the apex (Figure 2D). However, the 2C06 epitope residues were mostly conserved, with only a few residues having significant entropy, such as H85, E87, G644, and E648 (Figure 2E). Most residues of the fusion peptide were well conserved except for I515 and V518, which have moderate entropy.

Overall, the cryo-EM structure of 2C06 in complex with BG505 DS-SOSIP revealed 2C06 to bind at the fusion-peptide site of vulnerability, with heavy-chain CDRs accounting for most of the interactions with the fusion peptide.

### Cryo-EM structure of N751-2C09.01 in complex with BG505 DS-SOSIP reveals epitope similarity to N751-2C06.01

**Figure 3.**
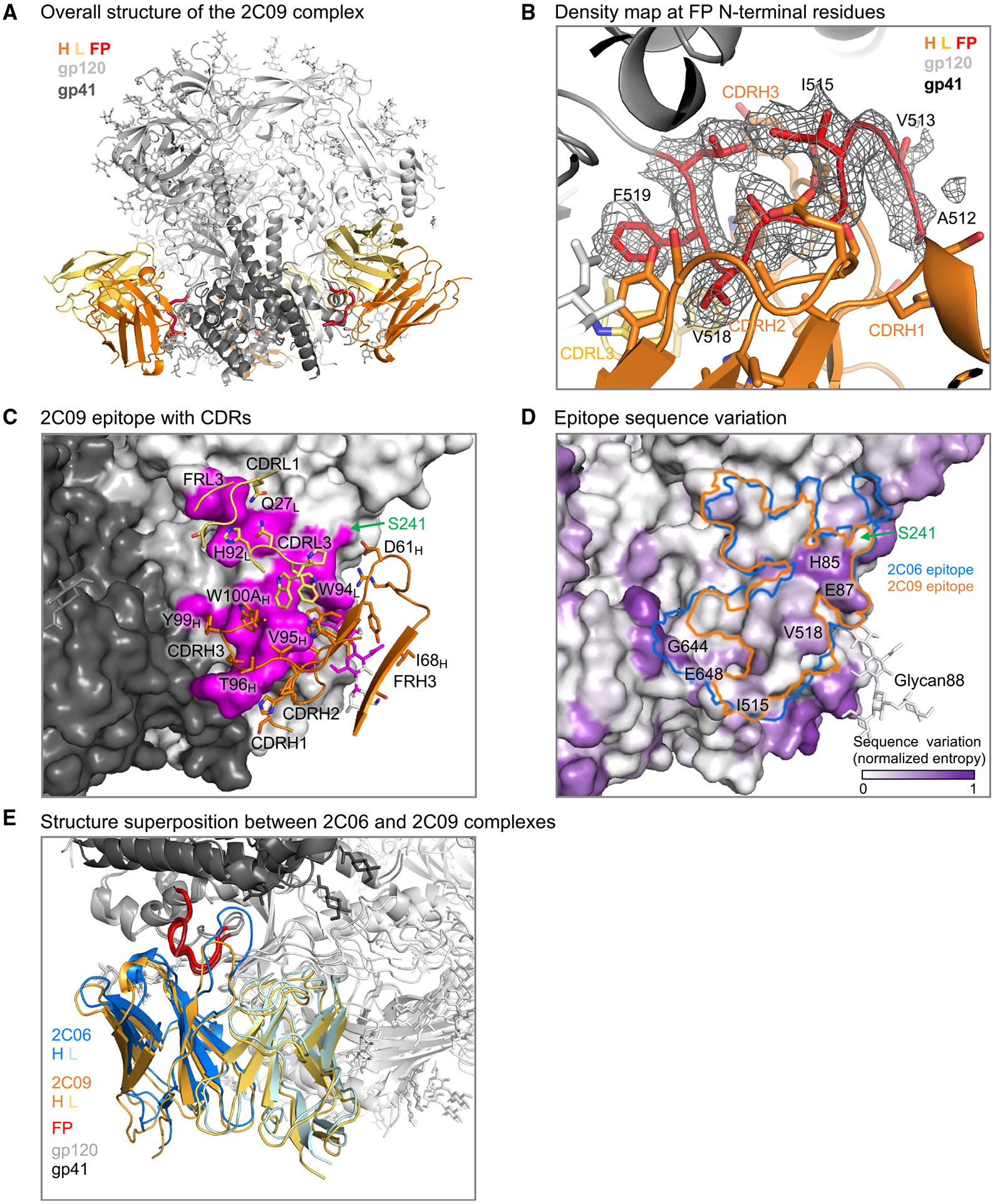
Cryo-EM structure of N751-2C09.01 in complex with BG505 DS-SOSIP reveals epitope similarity to N751-2C06.01 (A) Cartoon representation of 2C09 Fab in complex with BG505 DS-SOSIP. 2C09 bound at the fusion-peptide site and interacted with fusion peptide extensively. Heavy chain (H) is shown in orange, light chain (L) in yellow-orange, fusion peptide (FP) in red, the rest of gp41 in darker gray, and gp120 in lighter gray. (B) Density map showing well-ordered density for fusion peptide. The structure is shown in cartoon representation, with side chains of fusion-peptide residues 512–520 shown in sticks. Some side chains of the antibody interacting with fusion peptide are also shown in sticks. (C) Epitope details of antibody 2C09 shown for one of the binding sites on the trimer. Env trimer is shown as surface, with subunits in different shades of gray and the epitope surface in magenta. Antibody CDRs and FRs involved in binding are shown in cartoon representation, with interacting side chains shown in sticks. The glycan hole at S241 is on the edge of the epitope and is labeled. (D) Sequence conservation around the 2C09 epitope. Normalized sequence entropy of HIV-1 group M is mapped on the trimer surface, with absolutely conserved residues (entropy of 0) in white and the most diverse residues (entropy of 1) in purple. 2C09 epitope is shown as an orange outline. The outline of 2C06 is shown in blue for comparison. (E) Structure superposition between 2C06 and 2C09 complexes. The two structures were aligned by the gp41 subunit bearing the fusion peptide shown. The fusion peptide in both structures were in similar conformation except the first two residues, which in 2C09 (darker red) folded back down to interact with the antibody and in 2C06 extended up to bind the C-terminal helix of neighboring gp41 (top left). See also Figure S4 and Tables S1-S3.

To understand the differences and similarities between antibodies 2C06 and 2C09, we determined the cryo-EM structure of 2C09 in complex with BG505 DS-SOSIP. 2C09 binding to the envelope trimer did not induce noticeable asymmetry, and we obtained a 3D-reconstruction map at 2.81 Å with C3 symmetry from 445,733 particles (Figure S4 and Table S1). Like 2C06, 2C09 bound at the fusion-peptide site of vulnerability (Figure 3A), with 90% overlap of the two epitopes.

The fusion peptide in the 2C09 bound complex also formed a U-shape conformation, except for its two N-terminal residues (512 and 513), which turned back to interact with antibody. As a result, fusion-peptide binding to 2C09 involved residues 512–520 interacting almost exclusively with heavy-chain CDRs (Figure 3B), with ~470 Å^2^ of BSA (Tables S3 and S4). 2C09 interacted more extensively than 2C06 with the N-terminal residues of fusion peptide, providing an explanation for the stronger observed binding to shorter peptides by 2C09 versus 2C06 (Figure S2C). The 2C09 CDR L3 interacted with fusion-peptide residues 518 and 519, with ~90 Å^2^ BSA, from two tryptophan side chains, W94 and W96. The BSA with fusion peptide from CDRs of heavy and light chains together accounted for more than half of the total protein BSA of ~990 Å^2^ (not including BSA from the saccharide moiety at residue 88), a little less than that of 2C06.

The heavy chain of 2C09 contributed about 70% of the total epitope surface. The CDR H3 of 2C09 was four residues shorter than that of 2C06 and, unlike in the 2C06-Env complex, did not reach the neighboring gp41; this difference accounted for most of the difference in total BSA (Figures 3C and 3D). As such, the 2C09 epitope did not include residues G644 and E648, two of the high-entropy residues recognized by 2C06 on the neighboring protomer, but did include residues H85 and E87 (Figure 3D). Similar to 2C06, light-chain CDRs L1 and L3, and FR L3, were involved in trimer binding (Figure 3C), and most of the light-chain interactions were with residues 79–88. Binding to these residues was shared with the heavy chain and altogether accounted for ~280 Å^2^ BSA; interaction with glycan88 contributed an additional ~215 Å^2^ of heavy-chain BSA (Tables S3 and S4). The 2C09 epitope also recognized the S241 glycan hole (Figures 3C and 3D), in a manner similar to that of 2C06. Overall, the structure of 2C09 in complex with BG505 DS-SOSIP revealed a binding mode akin to that of 2C06 but with slightly reduced BSA due to a shorter CDR H3 (Figure 3E).

### Antibodies 2C06 and 2C09 form a reproducible antibody class

**Figure 4.**
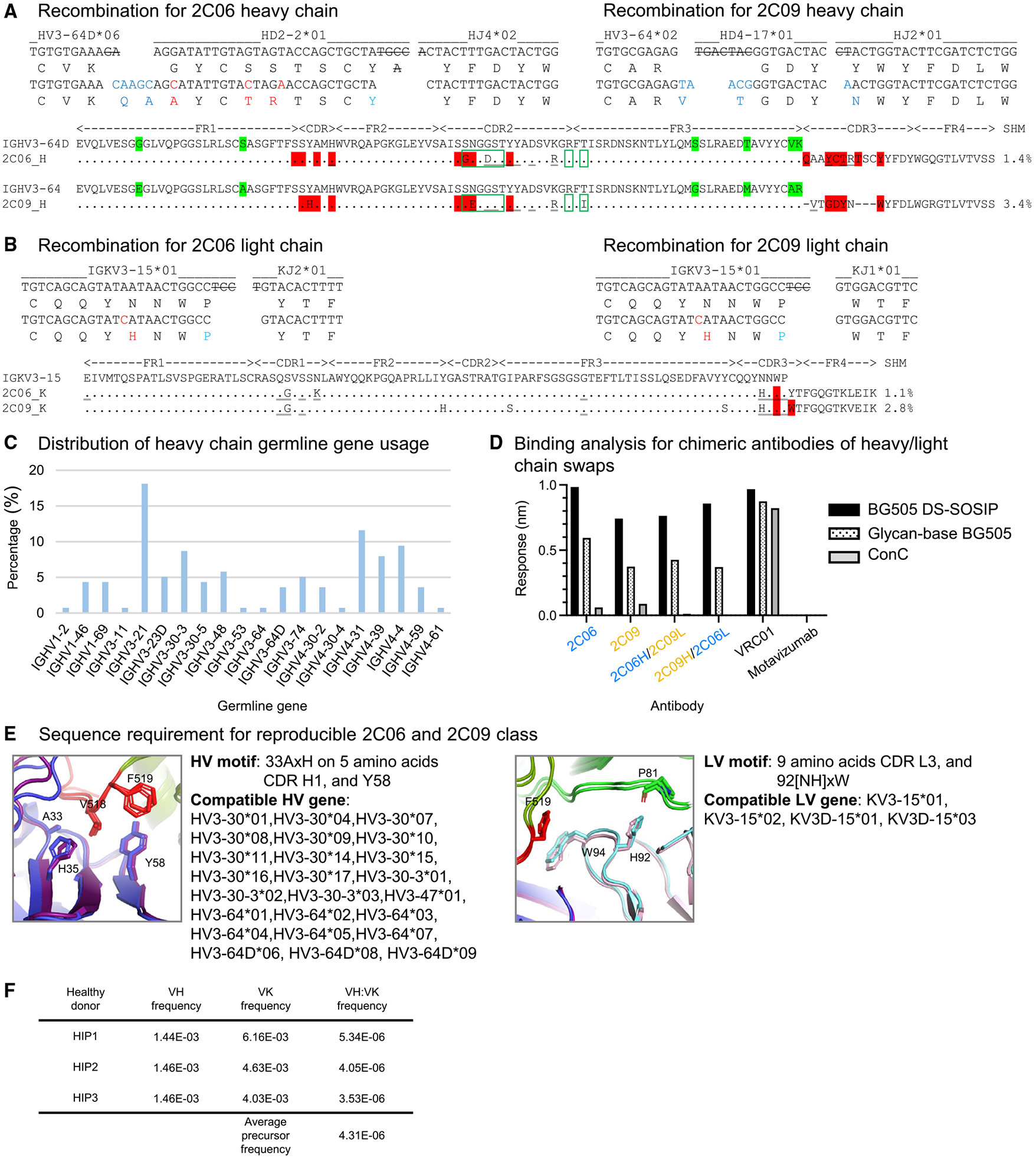
Antibodies 2C06 and 2C09 form a reproducible class (A and B) Analysis of junction regions and sequence alignment of 2C06 and 2C09. Germline gene nucleotide and amino acid residues are shown in black. The somatic hypermutations are colored red. Nucleotides removed by exonuclease trimming are crossed out. Germline amino acid differences between IGHV3-64 and IGHV3-64D are labeled with green highlight. Fusion-peptide contacts (red highlight), glycan contacts (green rectangles), and additional trimer contacts (underlined) are highlighted. (C) Heavy-chain germline gene usage of 138 antibodies isolated from donor N751. (D) Binding analysis of chimeras swapping heavy and light chains between 2C06 and 2C09. Data were measured once. (E) Sequence signatures of the reproducible 2C06 and 2C09 class for fusion-peptide binding. HV, heavy-chain variable; LV, light-chain variable. See also Table S6. (F) Calculated precursor frequency of 2C06/2C09 antibody class for healthy donors HIP1–HIP3 (see STAR Methods).

Antibodies 2C06 and 2C09 originated from similar heavy-chain variable genes, IGHV3-64D and IGHV3-64, respectively (Figures 4A and 4B). However, their heavy-chain D and J genes were different, indicating these two antibodies to be from two distinct lineages and resulting in different CDR H3 lengths. Among the 138 sequenced antibodies isolated from donor N751 using Env-trimer probes, IGHV3-64D accounted for ~4%, whereas IGHV3-64 accounted for <1% (Figure 4C and Table S6), indicating this similarity in V-gene usage to be unlikely to occur randomly; indeed, none of the other isolated antibodies that also bound both BG505 DS-SOSIP and glycan-base BG505 Env trimers derived from these heavy-chain V genes (Figures 1C and 1D; Table S6).

The similarity in origin genes and in Env-trimer recognition suggested that 2C06 and 2C09 might be members of a reproducible antibody class. Indeed, most residues involved in trimer binding were identical between 2C06 and 2C09, discounting the different length in CDR H3. Both 2C06 and 2C09 also showed low levels of somatic hypermutation (1% and 3%, respectively, based on nucleotide sequence) (Figures 4A and 4B).

To confirm whether 2C06 and 2C09 were members of the same antibody class, we swapped the heavy and light chains of the two antibodies, as such swapping has in the past confirmed class membership.^27-29^ The chimeric antibodies expressed well in mammalian cells and could be purified like the parental 2C06 and 2C09 antibodies. Bio-layer interferometry (BLI) analysis showed that the chimeras could bind BG505 DS-SOSIP and base-covered BG505, similar to 2C06 and 2C09 (Figure 4D). Informatics analysis of the structures revealed that 2C06 and 2C09 shared a heavy-chain variable motif consisting of A^33^XH (superscript number indicates the residue number for a multiple residue motif) and Y58 and a light-chain variable motif consisting of [NH]^92^XW for fusion-peptide binding, and these sequence motifs were compatible with multiple heavy-chain variable genes and several light-chain variable genes (Figure 4E). We used OLGA to calculate the precursor frequency for 2C06 and 2C09 (Figure 4F), finding a frequency of about ~1 in 232,000, which is higher than the estimated VRC01 class-antibody precursor frequency.^28^ Collectively, these results indicated that 2C06 and 2C09 formed a reproducible antibody class, and we provide details of the expected frequency of this class.

### Antibody 2C06 partially accommodates, but antibody 2C09 clashes with, glycan241

We next investigated the parameters governing breadth of recognition by antibodies 2C06 and 2C09. As described in the aforementioned structural analysis, both 2C06 and 2C09 epitopes involved S241, a rare glycan hole in BG505 among HIV isolates; specifically, in both 2C06 and 2C09, heavy-chain residues D61 and R64 were within 4–5 Å from S241 side chain, and PISA analysis indicated both 2C06 and 2C09 to bury 14–22 Å^2^ surface area with S241 (Table S4). However, the extent that the presence of a glycan at residue 241 might impact recognition was unclear from this analysis.

**Figure 5.**
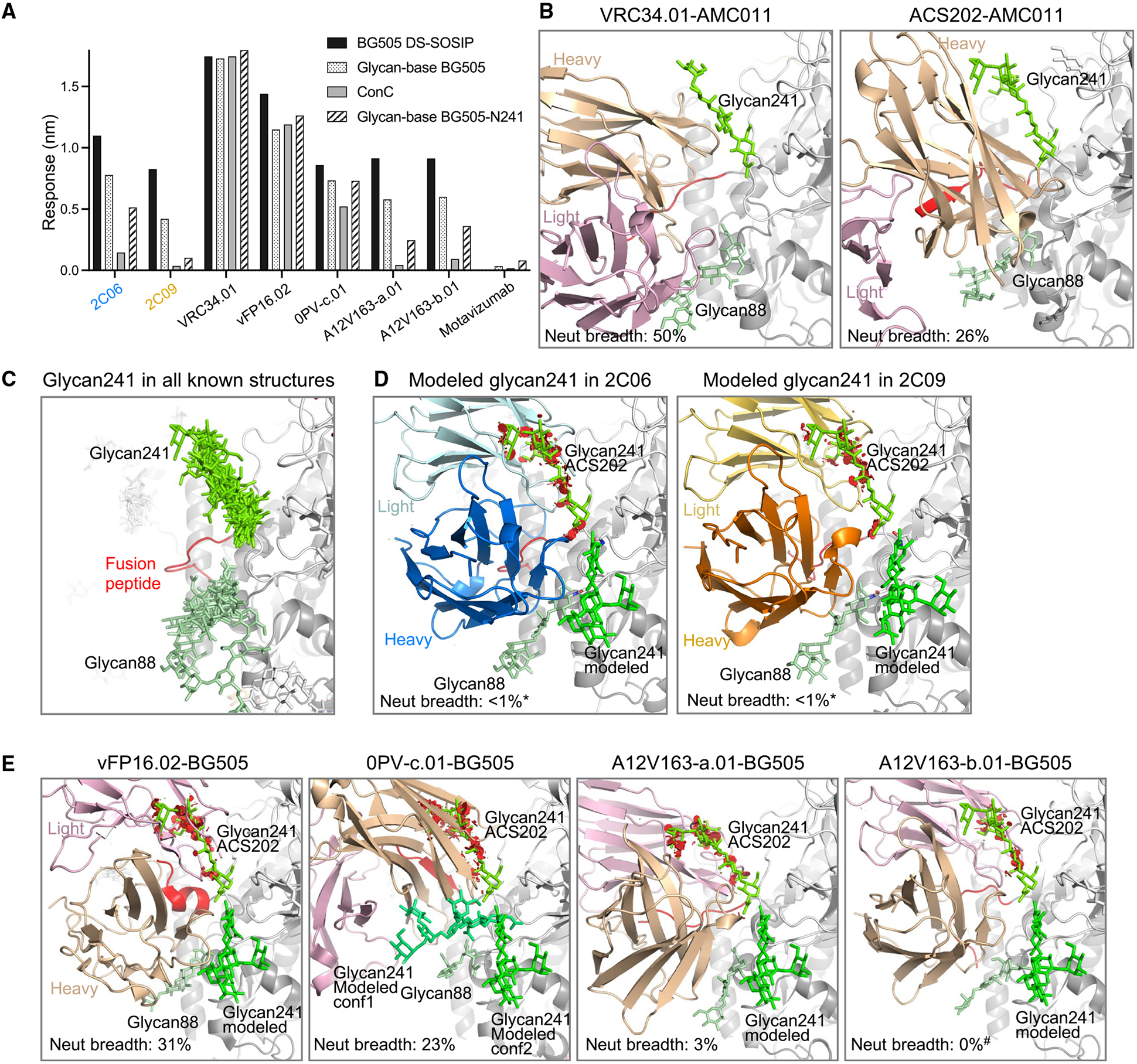
Antibody 2C06 partially accommodates, but antibody 2C09 clashes with, glycan241 (A) BLI binding analysis of 2C06 and 2C09 with Env trimers with or without glycan241 in comparison with other fusion-peptide-directed antibodies. Both BG505 DS-SOSIP and glycan-base BG505 have serine at 241. Glycan-base BG505-N241 is an S241N mutant of glycan-base BG505 containing *N*-glycosylation sequon at 241. ConC harbors glycan241. Motavizumab is a negative control. Data were measured once. (B) Structures of VRC34.01 (PDB: 6nc3) and ACS202 (PDB: 6nc2), both in complex with AMC011 Env, which contains glycan241, reveal that both antibodies interact favorably with glycan88 and glycan241, shown in different shades of green. Neutralization breadths were for half-maximal inhibitory concentration (IC_50_) < 50 μg/mL in the 208-strain panel. (C) Structural alignment of Env trimers from the PDB with two or more sugar residues built for glycan241. The structures were aligned by the gp120 subunit bearing the glycan241 shown. Antibodies, if present in the structure, are not shown for clarity. Glycan88 and glycan241 are shown in different shades of green, with the same view as in other structural panels of the figure. PDB: 6olp, 6vo3, 7rsn, 7rso, 6ohy, 7n6u, 6vrw, 6myy, 7llk, 6u59, 5vn8, 6p65, 6vy2, 7lua, 6okp, 7l6o, 6nc3, 6nc2. (D) Antibodies 2C06 and 2C09 clash with the glycan241 conformation seen in all known Env structures containing glycan241. The structures were aligned with that of ACS202-AMC011 complex by the gp120 subunit bearing the glycan241 shown. Clashes between antibody 2C06 or 2C09 and glycan241 in the ACS202 complex are shown as red disks in PyMOL (https://pymol.org/2/). A different glycan conformation could be modeled (shown in bright green) that did not clash with the antibody. Asterisks denote that 2C06 and 2C09 neutralize BG505 only. (E) Antibodies derived from mouse immunizations, vFP16.02 (PDB: 6cdi), and from non-human primates, 0PV-c.01 (PDB: 6nf2), A12V163-a.01 (PDB: 6n1v), and A12V163-b.01 (PDB: 6mgp), all of which clash with the glycan241 conformation in ACS202. A different glycan241 conformation, similar to that for 2C06 and 2C09, could be modeled, which avoids any clashes. Binding of 0PV-c.01 allows for more space for multiple conformations of modeled glycan241. Hash mark denotes that A12V163-b.01 neutralizes only at IC_50_ > 50 mg/mL. See also Figures S2 and S5; Table S4.

We therefore created an S241N mutant of glycan-base BG505 (glycan-base BG505-N241), which introduced *N*-linked glycan at position 241, and assessed binding by BLI to 2C06 and 2C09. We observed that presence of glycan241 substantially reduced the binding of 2C09 (to a level similar to the non-neutralizing motavizumab control), whereas 2C06 retained over half of its binding (Figure 5A). Binding to ConC Env trimer, which contains glycan241, was negligible for both 2C06 and 2C09. In comparison, antibody VRC34.01 bound strongly to BG505 DS-SOSIP, ConC, glycan-base BG505, and glycan-base BG505-N241. Other broadly neutralizing fusion-peptide-directed antibodies, vFP16.02 and 0PV-c.01,^17,30^ also retained a similar level of binding in the presence of glycan241. By contrast, the narrow-breadth antibodies, A12V163-a.01 and A12V163-b.01 elicited by trimer-only immunization in non-human primates,^17^ exhibited about half the reduction in binding in the presence of glycan241, similar to that observed for 2C06. Overall, the results indicated that binding by antibody 2C06 could partially accommodate the presence of a glycan at position 241, whereas antibody 2C09 clashed with glycan241.

To understand the roles of glycan241 in the recognition of Env trimers by antibodies, we compared the known structures of fusion-peptide-directed antibodies in complex with Env trimers. Except for PGT151,^31^ which bound at a site next to glycan611 and glycan637, most fusion-peptide-directed antibodies bound at a similar location, interacting favorably with glycan88 but variably with glycan241 (Figure S5). ACS202 and VRC34.01, both in complex with AMC011 Env,^32^ which contains glycan241, interacted favorably with both glycan88 and glycan241 (Figures 5B and S5B). The glycan241 conformations in these two structures are similar. In fact, alignment of all Env-trimer structures from the Protein Data Bank (PDB) with two or more sugar residues built for glycan241 revealed all glycan241 conformations to be similar (Figure 5C), suggesting this conformation to be the most energetically favorable. When this glycan241 conformation was superimposed on the structures of 2C06 and 2C09, however, steric clashes were observed (Figure 5D), although another glycan241 conformation could be modeled that avoided clashes with 2C06 and 2C09 (Figure 5D). We note that neutralizing antibodies vFP16.02, 0PV-c.01, A12V163-a.01, and A12V163-b.01 all showed substantial clashes with glycan241 in the conformation defined by antibody-ACS202 complexes (Figure 5E). The broadly neutralizing antibodies vFP16.02 and 0PV-c.01 likely accommodate the presence of 241, as they neutralize strains that have a glycan at this position; however, the strain-specific antibodies A12V163-a.01 and A12V163-b.01—although they retain at least one-third of their affinity to BG505-241—were incapable of neutralizing any strains with glycan241, suggesting that these antibodies, like 2C06 and 2C09, have their neutralization substantially impacted by glycan241. Notably, introduction of *N*-linked glycan at 241 in BG505 pseudovirus resulted in the loss of neutralization by both 2C06 and 2C09 (Figure S2E), confirming the importance of this glycan hole in the recognition of these two antibodies.

### High-entropy epitope residues contribute to the strain specificity of 2C06 and 2C09

While recognition of the 241 glycan hole explains much of the strain specificity of 2C06 and 2C09, there were seven strains other than BG505 in our 208-isolate panel missing glycan241, and we next investigated whether HIV sequence diversity of the epitope residues contributed to the strain-specific neutralization of these antibodies.

**Figure 6.**
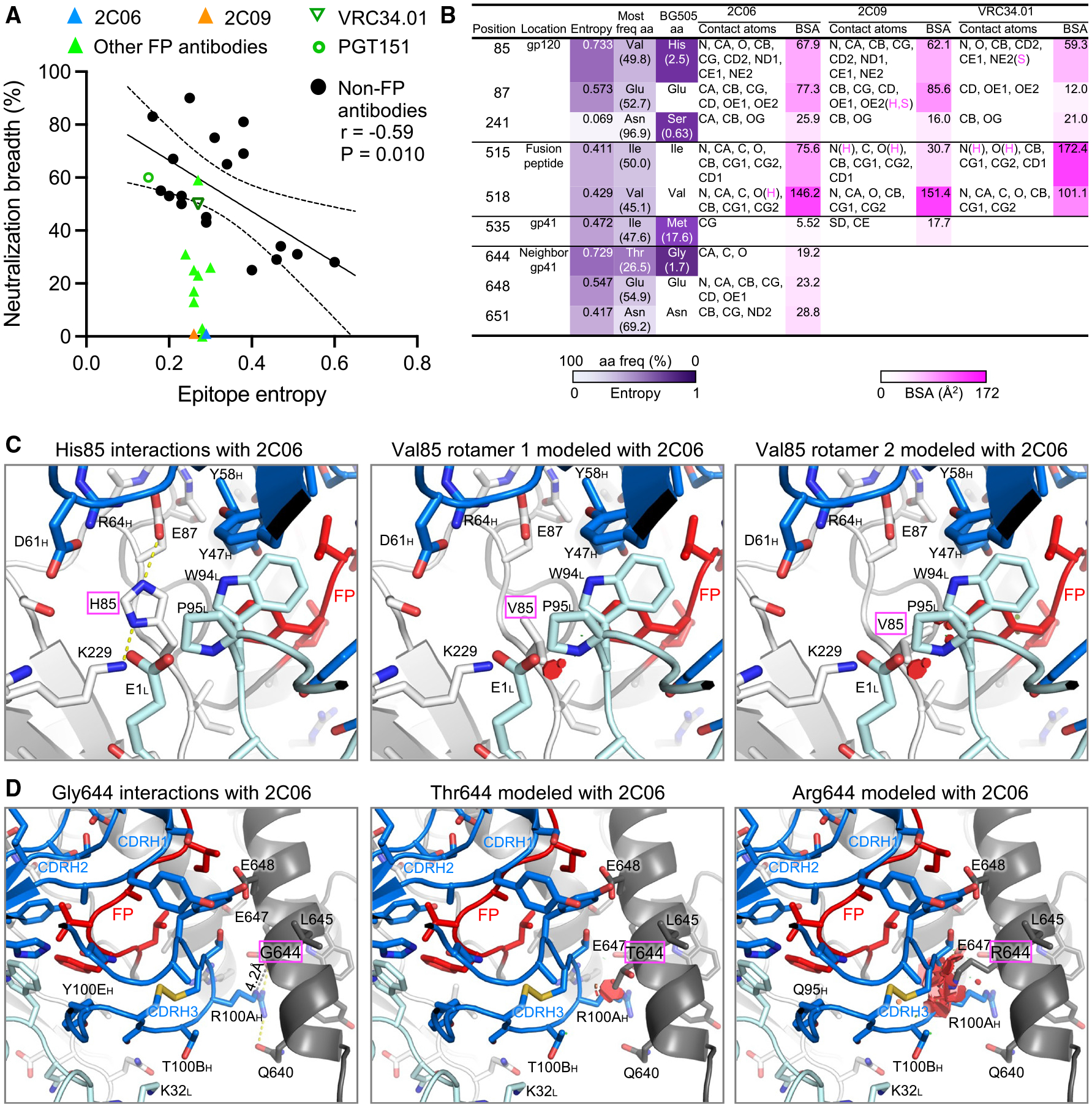
Recognition requirements for high-entropy residues contribute to strain specificity of 2C06 and 2C09 (A) While neutralization breadth generally correlates with epitope entropy, antibodies directed to the fusion-peptide sites of vulnerability have similar epitope entropy. Neutralization breadth represents the percentage of the 208-strain panel neutralized with IC_50_ < 50 μg/mL. Epitope entropy was calculated as BSA-weighted average of normalized Shannon’s entropy on the Env sequences of the 208-strain panel. (B) Analyses of sequence entropy and contact surface areas of residues in strain-specific and broadly neutralizing fusion-peptide-directed antibodies. Epitope residues with normalized entropy above 0.4 are listed, as well as residue S241, which has low entropy but with low frequency as serine among HIV-1 isolates. The contact atoms and areas for VRC34.01 were calculated from PDB: 5i8h (VRC34.01 also binds AMC011 Env, which has valine85). Entropy values were calculated with Shannon Entropy-One (https://www.hiv.lanl.gov/content/sequence/ENTROPY/entropy_one.html) on the curated alignment of year 2020 HIV-1 M group Env of 5,255 sequences (https://www.hiv.lanl.gov/content/sequence/NEWALIGN/align.html), and were normalized to have values between 0 and 1. Contact atoms and BSA were calculated with PISA (https://www.ebi.ac.uk/msd-srv/prot_int/cgi-bin/piserver). Hydrogen bonds or salt bridges involved with the contact atoms are designated as H or S, respectively. (C) H85, with high Shannon entropy of 0.733 and 2.5% frequency, has tight interactions with antibody heavy- and light-chain residues in both structures of the 2C06 complex (2C09 has nearly identical interactions, see Figure S6). Modeling in PyMOL with the most frequent amino acid valine results in clashes with CDR L3, either P95 or W94 or both, with any rotamers. The second most common amino acid at 85 is glutamate at 8.5% frequency and would have severe clashes with the antibody. (D) Antibody 2C06 had close contact with G644 from its R100A side chain of CDR H3, which played a major role in binding fusion peptide. Modeling as threonine (26.5% frequency) or arginine (20.1%) would result in clashes, disrupting CDR H3 interactions with fusion peptide. See also Figures S2 and S6.

We calculated the BSA-weighted average entropy of the epitope residues for fusion-peptide-directed antibodies as well as other broad HIV-neutralizing antibodies. We observed a negative correlation between epitope entropy and neutralization breadth for non-fusion peptide HIV-neutralizing antibodies (Figure 6A and Table S2). However, antibodies binding at the fusion-peptide site of vulnerability all had very similar normalized epitope entropies of 0.24–0.30, except for PGT151, which bound to a slightly different region from other fusion-peptide-directed antibodies (Figure S5). This finding suggested that the strain specificity for fusion-peptide-directed antibodies was unrelated to overall epitope entropy but instead related to an inability to accommodate the variation that is intrinsically inherent to the fusion-peptide site of vulnerability.

To identify epitope residues in BG505 that contribute to strain specificity, we analyzed Shannon entropy and BSA of epitope residues of 2C06 and 2C09 (Figure 6B). Fusion-peptide residues were well conserved among HIV isolates except at positions 515 and 518, with normalized entropy above 0.4 (Figure S6A). Outside of the fusion peptide itself, however, there were several residues in 2C06 or 2C09 epitopes with high entropy (Figure 6B). Of these, position 85 had a normalized entropy of 0.733, and in BG505 this position was a histidine, which had a frequency of 2.5% among HIV-1 group M isolates; and position 644 was a glycine in BG505, which had 1.7% frequency and a normalized entropy of 0.729. H85 had similar tight interactions with both 2C06 and 2C09, surrounded by side chains from CDR H2 (Y47 and Y58), FR H3 (D61 and R64), CDR L3 (W94 and P95), and light-chain N-terminal residue E1 (Figures 6C and S6B). The presence of the most frequent residue valine at this position would likely clash with CDR L3, which also played a role in binding fusion peptide (Figure 6C). In terms of neutralization, of the eight strains in our 208-strain panel lacking glycan241, there were two strains with histidine at 85, T257-31 and CNE56, in addition to BG505; both 2C06 and 2C09 could not neutralize these two strains (Figure S2E). Residue G644 in the 2C06 complex had tight interactions with R100A side chain of CDR H3, which played an important role in binding fusion peptide. Mutation of G644 to other amino acids would clash with arginine at residue position 100A, contributing to the strain-specific neutralization of 2C06 (Figure 6D). In terms of neutralization, other than BG505, none of the other strains lacking a glycan at 241 had G644, so 2C06 would be unlikely to neutralize these. 2C09 does not interact with position 644 and might have a chance to neutralize some of them. However, experimental assessment of neutralization indicated that neither 2C06 nor 2C09 could neutralize any of the other strains lacking a glycan at 241 in our 208-strain panel (Figure S2E).

To confirm the observed interactions of the epitope residues with substantial entropy, we mutated H85 or G644 in BG505 DS-SOSIP to the more frequent utilized amino acids among HIV-1 isolates and assessed the mutants for binding to 2C06 and 2C09 (Figure S6D). Mutation of H85 to valine, the most frequent amino acid, reduced the binding to both antibodies by half, whereas mutation to glutamate, the second most frequent, reduced binding by more than 6-fold. Mutation of G644 to arginine (the most frequent) or threonine (the second most frequent) reduced binding to 2C06 by more than 5-fold, but reduced 2C09 binding by about half. These results confirmed the role of H85 and G644 in antibody binding as observed in structures.

Overall, the above analyses suggested that fusion-peptide-directed neutralizing antibodies elicited by BG505 Env immunization can partially accommodate the glycan hole at position 241. Those that do, however, encounter other BG505 strain-specific residues, proximal to fusion peptide, and their inability to accommodate variation at these other positions contributes to their strain specificity.

## DISCUSSION

As noted above, the HIV Env trimer evades antibody-mediated neutralization by conformational masking, glycan shielding, and sequence variation. Env trimers stabilized in the prefusion-closed conformation, however, induce recognition of the neutralization-susceptible shape of Env, and their use in human vaccine trials such as VRC 018^22^ enables insight into how the two remaining mechanisms of Env evasion, glycan shielding and sequence variation, impact the induction of neutralizing responses. Here, we observed glycan shielding to reduce immunogenicity of most of the Env surface such that ~90% of Env-trimer reactive B cells were directed at the protein-exposed base of the trimer, and—for those antibodies that recognized other regions on the trimer—only ~1% bound with sufficient affinity to neutralize (Figure 1). In terms of sequence variation, the few elicited neutralizing antibodies showed partial recognition of strain-specific glycan holes, such as at glycan241 on BG505 (Figures 2, 3, 4, and 5), and these antibodies were generally incapable of accommodating sequence variation even at the moderate level (epitope entropies of 0.24–0.30) that appeared to be present in antibodies that target the fusion-peptide site of vulnerability (Figure 6). Thus, partial recognition of a BG505-specific glycan hole and binding requirements for a few BG505-specific residues combined to make antibodies 2C06 and 2C09 highly specific for strain BG505.

We previously observed in the VRC 018 clinical trial^22^ that some participants showed detectable neutralization against BG505.N611Q, a mutant BG505 strain with glycan611 removed that is >10-fold more sensitive to fusion-peptide-directed antibodies than wild-type BG505.^8^ This, along with the observation in the current study that the initially identified neutralizing antibodies from humans vaccinated with BG505 DS-SOSIP trimer were fusion-peptide directed, suggested that strain BG505 might be especially good at eliciting fusion-peptide-directed antibodies, likely because of the lack of glycan241, which is proximal to the fusion-peptide site of vulnerability, as the presence of a glycan hole increases the elicitation of antibodies targeting proximal epitopes.^33-35^

It will be interesting to see how prefusion-stabilized Env-trimer immunization can be altered to overcome glycan shielding and sequence variation. One way to overcome immunogenicity issues related to glycan shielding might be to mask the exposed protein base on the trimer with *N*-linked glycans^36^ or by using membrane-bound trimers, such as those delivered by liposomes^37,38^ or on virus-like particles.^39^ In terms of sequence variation, immunization with diverse trimers appears to be one way to avoid or to mature responses to strain-specific glycan holes. These strategies can be combined with epitope focusing such as by removal of glycans proximal to the CD4-binding site^34,40^ or by induction of antibodies against a conserved portion of an epitope, such as by first priming with the N terminus of the fusion peptide to create an antigenic hotspot.^17^ More recent results with non-human primates suggest that appropriate maturation of the immune response to the prefusion-stabilized Env trimers might be critical, with broadly neutralizing responses observed even after immunization with only a single Env trimer.^19^ It will be fascinating to determine the molecular characteristics of elicited neutralizing antibodies from this recent non-human primate study and to find out how they differ from those described here from Env-trimer immunization of humans.

### Limitations of the study

This study describes antibodies isolated from a single donor at a single time point. The quality of isolated antibodies is also limited by the source of the study, the VRC 018 clinical trial, which was designed for the primary goal of testing the safety of BG505 DS-SOSIP adjuvanted by alum as a vaccine candidate. As a result, the antibodies isolated have limited somatic hypermutations and low neutralization potency with strain specificity.

## STAR★METHODS

### RESOURCE AVAILABILITY

#### Lead contact

Further information and requests for resources and reagents should be directed to and will be fulfilled by Peter D. Kwong (pdkwong@ nih.gov).

#### Materials availability

Plasmids generated in this study are available upon request.

#### Data and code availability

- Cryo-EM maps have been deposited to the EMDB with accession codes EMD-29725 and EMD-29731, and fitted coordinates have been deposited to PDB with accession codes 8G4M and 8G4T.
- This paper does not report original code.
- Any additional information required to reanalyze the data reported in this paper is available from the lead contact upon request.

### EXPERIMENTAL MODEL AND SUBJECT DETAILS

#### Serum samples

The samples were collected under the Vaccine Research Center’s (VRC), National Institute of Allergy and Infectious Diseases (NIAID), National Institutes of Health protocol VRC 018 (NCT03783130) in compliance with the NIH Institutional Review Board (IRB) approved protocol and procedures. All subjects met protocol eligibility criteria and agreed to participate in the study by signing the NIH IRB approved informed consent. Research studies with these samples were conducted by protecting the rights and privacy of the study participants.

#### Cell lines

HEK293F, Expi293F and FreeStyle 293-F cells were purchased from Thermo Fisher Scientific. The cells were used directly from the commercial sources following manufacturer suggestions as described in detail below.

### METHOD DETAILS

#### Preparation of Env trimers and antigen-specific probes

BG505 DS-SOSIP and ConC trimers were expressed in stable CHO cell lines and purified by non-affinity chromatography.^21^ Glycan-base BG505 was stabilized in prefusion-closed conformation by structure-based stabilization and consensus repair.^59^ Biotinylated probes of BG505 DS-SOSIP, glycan-base BG505, ConC, and glycan-base BG505-N241 were expressed in 293Freestyle cells as a fusion protein with an N-terminal single-chain Fc tag cleavable by HRV3C digestion and an AVI-tag at C terminus.^60^ Cell supernatants were harvested, and the proteins were bound on a protein A column, washed, N-terminal Fctag cleaved, biotinylated, and eluted. The eluted proteins were passed through a Superdex 200 16/600 column with PBS, and the biotinylated proteins were conjugated to fluorescent streptavidin.

#### B cell sorting

Cryopreserved PBMC from VRC 018 clinical trial (ClinicalTrials.gov NCT03783130)^22^ at 2 weeks after third boost time point were thawed, treated with Benzonase nuclease (Millipore Corp.), washed with PBS and stained with viability dye Aqua Fluorescent Reactive (Invitrogen) for 2 min followed by staining with the following human antibodies: anti-IgM-BB700 (BD, customized), anti-CD21-PE594 (BD Biosciences, 563474), anti-CD20-APCH7 (BD Biosciences, 560734), anti-IgG-BUV3950 (BD Biosciences, 564229), anti-CD3-BV510 (BD Biosciences, 740187), anti-CD14-BV510 (Biolegend, 301842), anti-CD56-BV510 (BD Biosciences, 740171), anti-CD38-BUV661 (BD Biosciences, 612969), anti-CD19-BUV805(BD Biosciences, 749173), anti-IgD-BV570 (BD Biosciences, 624298), anti-CD27-BV605 (Biolegend, 302830) along with BG505-AF488, glycan-base BG505-PE and glycan-base BG505-APC probes diluted in Brilliant Stain Buffer (BD Biosciences, 563794) for 30 min at 4°C protected from light. Cells were washed with PBS containing 0.1% BSA twice and analyzed on FACS Symphony A5 (BD Biosciences) using Diva software. Cells were gated on live singlets CD3^−^ CD4^−^ CD14^−^ CD56^−^ IgD^+^ IgM^+^ CD19^+^ CD20^+^ IgG^+^ memory B cells and all glycan-base BG505 positive cells were single-cell sorted in 96-well plates coated with 5 μL of TCL buffer (Qiagen) containing 1% of 2-Mercaptoethanol (Sigma-Aldrich).

#### Rapid assembly, transfection, and production of immunoglobulins (RATP-Ig)

The sorted B cells were subjected to RATP-Ig following the procedures as described previously.^25^ Briefly, single-cell RNA was purified with RNAclean beads (Beckman Coulter). cDNA was then synthesized using 5′ RACE reverse-transcription and amplified by PCR, and heavy and light chain variable regions enriched. An aliquot of enriched cDNA was sequenced using 2 × 150 paired-end reads on an Illumina MiSeq. For immunoglubulin production, enriched variable regions were assembled into expression cassettes that include CMV, and HC/LC-TBGH polyA fragments. Assembled cassettes were amplified by PCR and transfected into Expi293 cells in 96-well deep-well plates using the Expi293 Transfection Kit (ThermoFisher Scientific). Cell cultures were grown at 37°C, 8% CO_2_, and 1100 RPM shaking for 5–7 days. Cell culture supernatants were harvested by centrifugation.

#### AlphaLISA screening of RATP-Ig supernatants

RATP-Ig supernatants from glycan-base BG505-positive B cells were diluted into AlphaLISA buffer (PBS +0.05% Tween 20 + 0.5 mg/mL BSA), and 5 μL of each were transferred to an OptiPlate-384 assay plate (white opaque, PerkinElmer, Waltham, MA). To each well was added 10 μL of biotinylated Env trimer probe at 10 nM final concentration and 10 μL of anti-human Fc IgG (PerkinElmer, Waltham, MA) acceptor beads at 10 μg/mL final concentration. The mixtures were incubated at room temperature (RT) for an hour and then added 25 μL of streptavidin donor beads (PerkinElmer, Waltham, MA) at 40 μg/mL final concentration. The plate was incubated at RT for 30 min in the dark, and the AlphaLISA signal was read using a SpectraMax i3x multi-mode microplate reader (Molecular Devices, San Jose, CA). Only nine supernatants out of two plates of RATP-Ig supernatants were identified as double-positive for binding BG505 DS-SOSIP and glycan-base BG505, likely due to both false positives in B cell sorting and low level of Ig expression in RATP-Ig for some antibodies.

#### Antibody preparation

DNA encoding antibody heavy- and light-chain variable regions were synthesized and cloned into the pVRC8400 vectors.^61^ Plasmids of heavy and light chain pairs were co-transfected in Expi293F cells (Thermo Fisher) using Turbo293 transfection reagent (Speed BioSystems) as described previously.^62^ On day 6 post transfection, the culture supernatant was harvested and loaded on a protein A column. The column was washed with PBS, and the IgG protein was eluted with a low pH buffer. For Fab preparation, an HRV3C cleavage site was inserted in the heavy-chain hinge region in the pVRC8400 vectors. The eluted IgG protein was digested with HRV3C, and the Fab was purified from a protein A column and then further purified from a Superdex 200 column (Cytiva).

#### Env-pseudovirus neutralization assays

Monoclonal antibody neutralization was assessed based on the single-round infection assay of TZM-bl cells with HIV-1 Env-pseudoviruses as described previously.^47^ Purified monoclonal antibodies were tested for neutralization against wild-type HIV-1 strains and the glycan mutants of BG505. Data were calculated as half-maximum inhibitory concentration (IC_50_) and 80% maximum inhibitory concentration (IC_80_) by comparison with control wells in the absence of antibodies.

#### Enzyme-linked immunosorbent assay (ELISA)

Anti-fusion peptide ELISA was performed by coating 96-well plates (Costar High Binding Half-Area; Corning, Kennebunk, ME) overnight at 4°C with 50 μL/well of 2 μg/mL FP8v1-rTTHC (AVGIGAVF), FP7v1-rTTHC (AVGIGAV), or FP6v1-rTTHC (AVGIGA) in PBS. Between each subsequent step, plates were washed five times with PBS-T (PBS plus 0.05% Tween) unless otherwise stated. Plates were blocked with 100 μL/well of in-house B3T blocking solution (30 mM NaCl, 10 mM Tris-HCl, 0.2 mM EDTA, 0.66% fetal bovine serum [FBS], 0.4% bovine albumin, 0.014% Tween 20, 0.004% thimerosal) for 1 h at 37°C. Next, 50 μL/well of serially diluted 2C06 or 2C09 antibody (7-point, 5-fold, 2 μg/mL starting concentration) were added to the plates and incubated for 1 h at 37^°^C. Goat anti-human IgG γ-chain horseradish peroxidase conjugated secondary antibody (Jackson Immunoresearch, West Grove, PA) diluted 1:5000 in B3T blocking solution was added to the plates (50 μL/well) and incubated for 1 h at 37°C. Next, plates were developed with 50 μL/well tetramethylbenzidine (TMB) substrate (SureBlue; KPL, Gaithersburg, MD) for 10 min at RT, and the reaction was stopped with 50 μL/well 1 N sulfuric acid (Fisher Chemical, Fair Lawn, NJ) without washing. Plates were read at 450 nm (SpectraMax using SoftMax Pro, Version 5, software; Molecular Devices, Sunnyvale, CA), and the optical densities (OD) were analyzed following subtraction of the non-specific horseradish peroxidase background activity.

Competition ELISA for antibody binding to BG505 DS-SOSIP was performed by coating 96-well plates (Costar High Binding Half-Area; Corning, Kennebunk, ME) overnight at 4°C with 50 μL/well of 2 μg/mL snowdrop lectin from *Galanthus nivalis* (Sigma-Aldrich, St. Louis, MO) in PBS. Between each subsequent step, plates were washed five times with PBS-T (PBS plus 0.05% Tween) unless otherwise stated. Following lectin coating, plates were blocked with 100 μL/well of blocking buffer (5% skim milk in PBS) (FisherScientific, Waltham, MA) for 1 h at RT. Blocking was followed by trimer capture with 50 μL/well of 2 μg/mL BG505 DS-SOSIP in FBS-PBS diluent (PBS plus 10% FBS) for 2 h at RT. Next, 50 μL/well mouse Fc competitor antibodies targeting several important trimer epitopes (fusion peptide, V1V2 region, CD4 binding site, V3 glycan, gp120/gp41 interface, or trimer base) were added at a fixed concentration of 2 μg/mL in blocking buffer and incubated for 1 h at RT. Without washing the plate, 50 μL/well of serially diluted 2C06 or 2C09 antibody (7-point, 5-fold, 2 μg/mL starting concentration) were added to the plates and incubated for 1 h at RT. Next, goat anti-human IgG γ-chain horseradish peroxidase conjugated secondary antibody (Jackson Immunoresearch, West Grove, PA) diluted 1:5000 in blocking buffer was added to the plates (50 μL/well) and incubated for 1 h at RT. Next, plates were developed with 50 μL/well TMB substrate (SureBlue; KPL, Gaithersburg, MD) for 10 min at RT, and the reaction was stopped with 50 μL/well 1 N sulfuric acid (Fisher Chemical, Fair Lawn, NJ) without washing. Plates were read at 450 nm (SpectraMax using SoftMax Pro, Version 5, software; Molecular Devices, Sunnyvale, CA), and the optical densities (OD) were analyzed following subtraction of the non-specific horseradish peroxidase background activity. Area under the curve (AUC) values of 2C06 or 2C09 were calculated from reciprocal dilution curves in the competition assay (GraphPad Prism 9.4.1 software). AUC values of 2C06 or 2C09 binding in the absence of competitor antibody represent unblocked trimer binding from which AUC values of 2C06 or 2C09 binding in the presence of competitor antibody were subtracted. This value was divided by unblocked trimer binding AUC and multiplied by 100 to obtain the percent reduction in trimer binding. Measurements were carried out in triplicates; mean and SE are reported.

#### IgG binding affinity measured by Carterra

A medium density HC30M sensor chip was preconditioned with 1-min pulses of each of three solutions: 50 mM NaOH, 1 M NaCl, and 10 mM Glycine pH 2.0. After preconditioning, the chip was activated with a 10-min injection of a freshly prepared 1:1:1 (v/v/v) mixture of 0.4 M 1-ethyl-3-(3-dimethylaminopropyl) carbodiimide hydrochloride (EDC), 0.1 M *N*-hydroxysulfosuccinimide (sNHS) and 0.1 M 2-(*N*-morpholino) ethanesulfonic acid (MES) pH 5.5. Then, 100 μg/mL of goat anti-human Fc antibody prepared in 10 μM sodium acetate pH 4.5 + 0.01% Tween 20 was coupled for 15 min and the excess reactive esters were blocked with 1 M ethanolamine HCl pH 8.5 during a 5 min injection. Antibodies were prepared at 10 mg/mL in running buffer (10 mM HEPES pH 7.4, 150 mM NaCl, 3 mM EDTA, and 0.01% (v/v) Tween 20 (HBSTE) supplemented with 0.5 mg/mL BSA) and captured for 15 min. The antigens (BG505 DS-SOSIP and glycan-base BG505) were then prepared at 0, 3.9, 7.8, 15.6, 31.2, 62.5, 125, 250, and 500 nM in HBSTE +0.5 mg/mL BSA and injected with an association time of 5 min, followed by a 15 min dissociation. Samples were injected in ascending concentration and the surface was regenerated with IgG elution buffer (glycine, pH 2.8) after finishing the concentration series of each antigen. Binding data from the local reference spots was used to subtract signal from the active spots and the nearest buffer blank analyte responses were subtracted to double-reference the data. The double-referenced data were fitted to a simple 1:1 Langmuir binding model in Carterra’s Kinetic Inspection Tool.

#### Bio-layer interferometry (BLI)

An Octet RED instrument (FortéBio) instrument was used to measure recognition of isolated antibodies to HIV-1 BG505 DS-SOSIP, glycan-base BG505, ConC and glycan-base BG505-N241 trimers. Isolated antibodies (50 μg/mL) were immobilized for 300 s on AHC biosensor tips and equilibrated for 60 s in Octet Kinetics Buffer (Sartorius Corporation) prior to measuring association with HIV-1 trimer diluted in Octet Kinetics Buffer (100 μg/mL). The association of HIV-1 trimers were recorded for 300 s. All BLI experiments were conducted at 30°C and were performed in duplicate. Parallel correction to subtract systematic baseline drift was carried out by subtracting the measurements recorded for a loaded sensor incubated in Octet Kinetics Buffer. Response levels of sensors were analyzed using Octet and GraphPad Prism 9 software.

#### Surface plasmon resonance measurements of Fab binding affinity

Binding affinities and kinetics of 2C06 and 2C09 antibodies were assessed by surface plasmon resonance (SPR) on a Biacore S-200 (GE Healthcare) at 25°C in HBS-P+ buffer (10 mM HEPES, pH 7.4, 150 mM NaCl and 0.05% surfactant P-20). Glycan-reactive 2G12 IgG was immobilized onto a CM5 chip by amine coupling to 8000–10000 response units (RU). BG505 DS-SOSIP at 25 nM were captured to the sample channel at 300 RU on the 2G12 sensor chip. Serial two-fold diluted 2C06 and 2C09 Fabs were passed through the sample and reference channels for 180 s followed by a 300 s dissociation phase at 30 μL/min. The surface was regenerated by flowing 3 M MgCl_2_ solution for 30 s at a flow rate of 50 μL/min. Blank sensorgrams were obtained by injection of the same volume of HBS-P+ buffer in place of antibody Fab solution. Sensorgrams of the concentration series were corrected with corresponding blank curves.

#### Cryo-EM sample preparation and data collection

Samples for cryo-EM grid preparation of the 2C06-BG505 DS-SOSIP complex were produced by first mixing 15 μL of purified BG505 SOSIP at 4 mg/mL with 55 μL of 2C06 Fab at 1 mg/mL; n-Dodecyl β-D-maltoside (DDM) was added to have a final concentration of 0.005% (w/v) to prevent preferred orientation and aggregation during vitrification, and the mixture was incubated on ice for 20 min. Cryo-EM grids were prepared by applying 3 μL of sample to a freshly glow discharged carbon-coated copper grid (CF 1.2/1.3 300 mesh). The sample was vitrified in liquid ethane using a Vitrobot Mark IV with a wait time of 30 s, a blot time of 3 s, and a blot force of 0. Cryo-EM data were collected using Leginon software^48^ on a Titan Krios electron microscope operating at 300 kV, equipped with a Gatan K3-BioQuantum direct detection device. Exposures were taken with a total electron fluence of 58.06 e−/Å^2^. The total dose was fractionated for 2.5 s over 50 raw frames.

For preparing the 2C09-BG505 DS-SOSIP complexes, Env was mixed with the antibody Fab at 1 to 1.2 M ratio at a final total protein concentration of ~3 mg/mL; DDM (1 mM stock solution) was added to a final concentration of 0.1 mM. 2.7 μL of the mixture was pipetted to a Quantifoil-gold 2/2 holey carbon grids, glow discharged for 30 s in a PELCO easiGlow Glow Discharge Cleaning System prior to cryo-grid preparation, and the grid was blotted for 2.5–4 s and plunge frozen into liquid ethane using a Vitrobot Mark IV (ThermoFisher). Cryo-EM data were collected on a Titan Krios operating at 300 kV, equipped with a K2 Summit detector (Gatan) operating in counting mode. Data were acquired using SerialEM 4.0.^63^ The dose was fractionated over 40 raw frames.

The movie frames were aligned and dose-weighted^50^ using cryoSPARC 3.3;^64^ and CTF estimation, particle picking, 2D classifications, *ab initio* model generation, heterogeneous refinements, homogeneous 3D refinements, non-uniform refinement, and local resolution calculations were carried out using cryoSPARC 3.3.

#### Model building and refinement

For structural determination, a model of the antibody Fab was generated using AlphaFold2 python notebook (https://colab.research.google.com/github/sokrypton/ColabFold/blob/main/beta/AlphaFold2_advanced.ipynb). The Fab model and the structure of BG505 DS-SOSIP (PDB: 6cdi)^30^ were docked into the cryo-EM density map using UCSF Chimera^51^ to build an initial model of the complex. The model was then manually rebuilt to the best fit into the density using Coot^52^ and refined using Phenix.^53^ Glycan structures were validated using Privateer and pdb-care.^54,55^ Alternative glycan conformations were modeled by Glycan Reader & Modeler of CHARMM-GUI.^65-68^ Overall structures were evaluated using MolProbity.^56,57^ Protein interface calculations were performed using PISA.^58^ Structural figures were generated using PyMOL (Schrödinger; http://www.pymol.org) and UCSF ChimeraX.^69^

#### Antibody recombination and sequence signature analyses

IgBlast was used to annotate antibody germline gene and to analyze V(D)J recombination event.^70^ The buried surface area (BSA) at the antibody-Env interface was calculated with PDBePISA.^58^ The structure-based process used to identify sequence signature for classifying antibodies was as previously described.^29^ Briefly, for antibodies with similar binding mode, we defined sequence motif by residues with a BSA value larger than 10 Å^2^. We compared sequence motifs that had similar recognition. Then, such sequence motifs with compatible VH/VL germline genes were selected as the sequence signature.

The precursor frequency was calculated using OLGA software.^71^ Three healthy donor NGS samples were used to learn the model.^72^ We used frequency of 60% for heavy chain pairing with kappa light chain (with a frequency of 40% for pairing with lambda light chains).^73^

### QUANTIFICATION AND STATISTICAL ANALYSIS

Cryo-EM data were processed and analyzed using CryoSparc. Cryo-EM structural statistics were analyzed with Phenix and Molprobity. Statistical details of experiments are described in method details or figure legends.

## Supplementary Material

1

2

## Figures and Tables

**Table:** KEY RESOURCES TABLE

REAGENT or RESOURCE	SOURCE	IDENTIFIER
Antibodies		
0PV-c.01	*Kong et al.* ^17^	N/A
N751-1B06	This study	N/A
N751-1C05	This study	N/A
N751-1C07	This study	N/A
1E6	*Cottrell et al.* ^41^	N/A
N751-1F10	This study	N/A
N751-2B06	This study	N/A
N751-2C06	This study	N/A
N751-2C09	This study	N/A
N751-2D02	This study	N/A
35O22	*Huang et al.* ^42^	N/A
3BNC117	*Scheid et al.* ^43^	RRID: AB_2491033
5H3	*Cottrell et al.* ^41^	N/A
A12V163-a.01	*Kong et al.* ^17^	N/A
A12V163-b.01	*Kong et al.* ^17^	N/A
Motavizumab	*Cingoz* ^44^	RRID: AB_2910856
PGDM1400	*Sok et al.* ^45^	N/A
PGT122	*Walker et al.* ^10^	RRID: AB_2491042
PGT151	*Blattner et al.* ^31^	N/A
vFP16.02	*Xu et al.* ^30^	N/A
VRC01	*Wu et al.* ^46^	RRID: AB_2491019
VRC34.01	*Kong et al.* ^8^	RRID: AB_2819225
Chemicals, peptides, and recombinant proteins		
Pierce Protein A Agarose	ThermoFisher Scientific	20334
Turbo293 transfection reagent	SPEED BioSystem	PXX1002
AbBooster medium	ABI scientific	PB2668
Bacterial and virus strains		
BG505	NIH/VRC	N/A
BG505.N611Q	NIH/VRC	N/A
BG505.S241N	NIH/VRC	N/A
Deposited data		
2C06-BG505 DS-SOSIP structure	This study	PDB: 8G4M
2C06-BG505 DS-SOSIP maps	This study	EMDB: EMD-29725
2C09-BG505 DS-SOSIP structure	This study	PDB: 8G4T
2C09-BG505 DS-SOSIP maps	This study	EMDB: EMD-29731
Experimental models: Cell lines		
293F Freestyle cells	Thermo Fisher	K900001
Expi293F cells	Thermo Fisher	A14527
Recombinant DNA		
pVRC8400-1B06 plasmids	This study	N/A
pVRC8400-1C05 plasmids	This study	N/A
pVRC8400-1C07 plasmids	This study	N/A
pVRC8400-1C07 plasmids	This study	N/A
pVRC8400-1F10 plasmids	This study	N/A
pVRC8400-2B06 plasmids	This study	N/A
pVRC8400-2C06 plasmids	This study	N/A
pVRC8400-2C09 plasmids	This study	N/A
pVRC8400-2D02 plasmids	This study	N/A
Software and algorithms		
Chimera	Pettersen et al.^47^	https://www.cgl.ucsf.edu/chimera/
ChimeraX	Pettersen et al.^48^	https://www.cgl.ucsf.edu/chimerax/
Coot	Emsley and Cowtan^49^	https://sbgrid.org/software/
CryoSparc	Punjani et al.^50^	https://guide.cryosparc.com/
IgBlast	Ye et al.^51^	https://www.ncbi.nlm.nih.gov/igblast/
Leginon software	Suloway et al.^52^	https://emg.nysbc.org/redmine/projects/leginon/wiki/Leginon_Homepage
MolProbity	Barad et al.; Williams et al.^53,54^	http://molprobity.biochem.duke.edu
OLGA	Sethna et al.^55^	https://github.com/statbiophys/OLGA
PDBePISA	Krissinel et al.^56^	https://www.ebi.ac.uk/pdbe/pisa/
Phenix	Adams et al.^57^	https://sbgrid.org/software/
PRISM	GraphPad Software	https://www.graphpad.com/scientific-software/prism/
Pymol	Schrödinger LLC	https://pymol.org
SerialEM 4.0	Mastronarde^58^	https://bio3d.colorado.edu/SerialEM/

## Highlights

- HIV-1 neutralizing antibodies 2C06 and 2C09 from VRC 018 clinical trial are isolated
- Cryo-EM structures of 2C06 and 2C09 in complex with HIV envelope trimer are determined
- 2C06 and 2C09 binding requires a glycan hole at 241 and BG505-specific residues
- Antibodies 2C06 and 2C09 form a reproducible class, recognizing fusion peptide
