# Supplementary material for: HIV-1 neutralizing antibodies elicited in humans by a prefusion-stabilized envelope trimer form a reproducible class targeting fusion peptide
Source: Cell Rep. Author manuscript; Available in PMC 2023 Sep 8. (PMC10491024; doi:10.1016/j.celrep.2023.112755)
Supplement: 1 [file NIHMS1920497-supplement-1.pdf]

Cell Reports, Volume *42*

Supplemental information

HIV-1 neutralizing antibodies elicited in humans by a prefusion-stabilized envelope trimer form a reproducible class targeting fusion peptide

Shuishu Wang, Flavio Matassoli, Baoshan Zhang, Tracy Liu, Chen-Hsiang Shen, Tatsiana Bylund, Timothy Johnston, Amy R. Henry, I-Ting Teng, Prabhanshu Tripathi, Jordan E. Becker, Anita Changela, Ridhi Chaudhary, Cheng Cheng, Martin Gaudinski, Jason Gorman, Darcy R. Harris, Myungjin Lee, Nicholas C. Morano, Laura Novik, Sijy O'Dell, Adam S. Olia, Danealle K. Parchment, Reda Rawi, Jesmine Roberts-Torres, Tyler Stephens, Yaroslav Tsybovsky, Danyi Wang, David J. Van Wazer, Tongqing Zhou, Nicole A. Doria-Rose, Richard A. Koup, Lawrence Shapiro, Daniel C. Douek, Adrian B. McDermott, and Peter D. Kwong

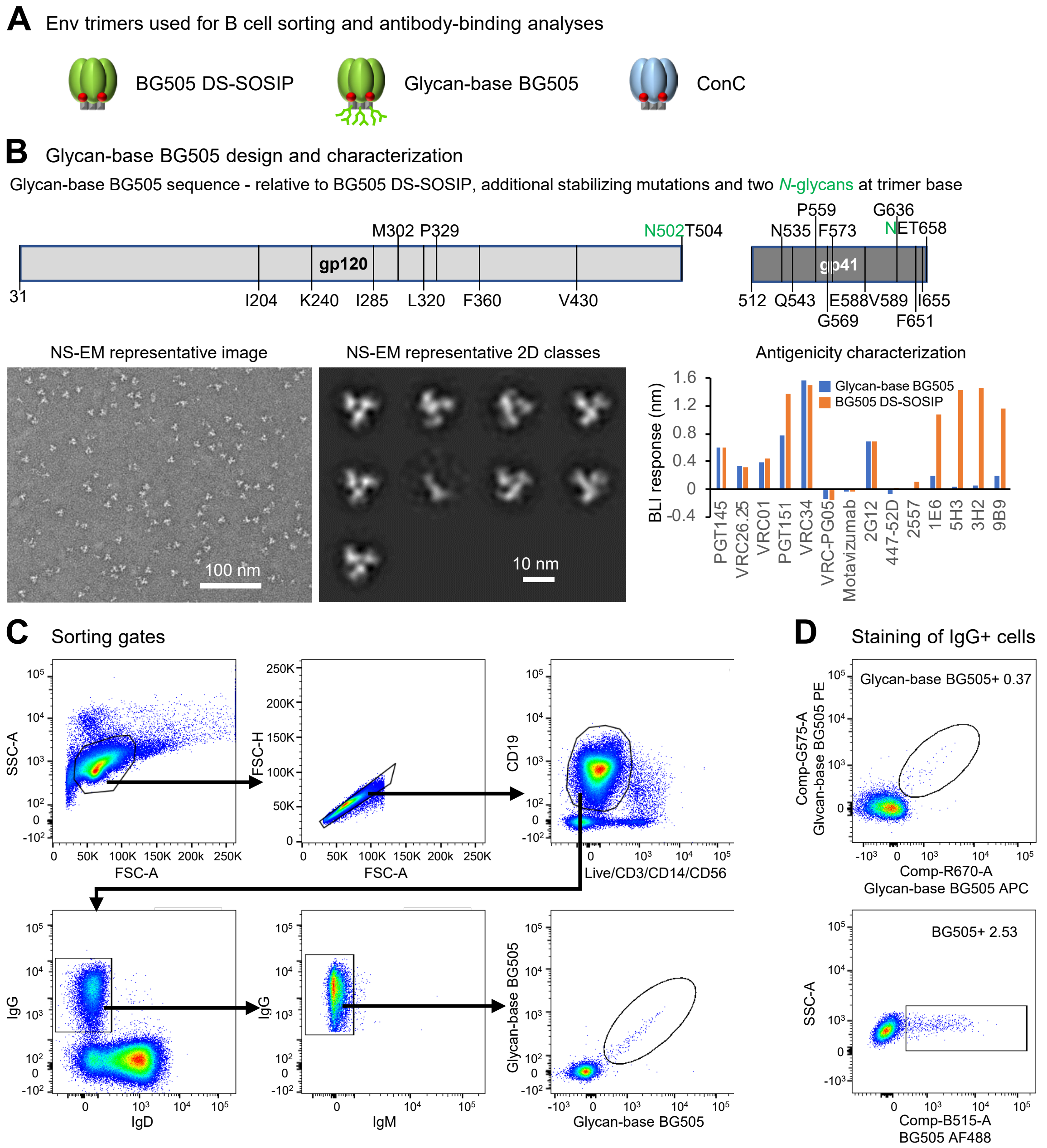

Figure S1. Env trimers and B cell sorting details, related to Figure 1.

(A) Env trimers used for B cell sorting and binding analyses with AlphaLISA, BLI, and SPR.

(B) The probe glycan-base BG505 was designed based on BG505 DS-SOSIP with additional mutations to stabilized the trimer in prefusion-closed conformation and two additional N-glycans to cover the trimer base. Negative stain (NS) EM revealed well-formed trimers, but with some monomeric shape present. Glycan-base BG505 bound most antibodies similarly to BG505 DS-SOSIP, except for the base-directed antibodies 1E6, 5H3, 3H2, and 9B9, to which it had negligible affinity.

(C) Details of B cell sorting gate strategy.

(D) Cytometry of IgG+ B cells from N751 serum. Cells were stained first with glycan-base BG505 to allow all non-base directed B cells to engage first in the trimer, washed the cells, and then stained with BG505 to allow the base-specific B cells to engage. BG505+ cell population is much larger than glycan-base BG505+ cell population. The glycan-base BG505-positive cells account for 0.37% of IgG+ memory B cells (Singlet, Live, CD19+, CD3-, CD14-, CD56-, IgG+) (upper panel). After absorption with glycan-base BG505, 2.53% of IgG+ memory B cells bind BG505 DS-SOSIP trimer (lower panel). Glycan-base BG505+ cells are 12.89% of all antigen-specific memory B cells, and the other 87.11% are likely targeting the trimer base.

A Pseudovirus neutralization assessments for 2D02 and 1B06

	IC50 μg/ml			IC80 μg/ml		
Viral strains	MW965.26	BG505.W6M.C2	BG505.W6M.C2.N611Q	MW965.26	BG505.W6M.C2	BG505.W6M.C2.N611Q
Clade	C	A		C	A	
2D02 IgG	>50	>50	>50	>50	>50	>50
1B06 IgG	>50	>50	>50	>50	>50	>50

B Neutralization assessments against a panel of viruses neutralized by most fusion peptide-directed antibodies

	Viral strains	BG505.W6M.C2.N611Q	BG505.W6M.C2	HxB2.DG	KER2008.12	MB201.A1	MI369.A5	96ZM651.02	CNE40	Q23.17	CH117.4	3468.V1.C12	3365.v2.c20	ZM135.10a	BI369.9A	0815.v3.c3	CNE19
	Clade	A	A	B	A	A	A	C	BC	A	BC	AD	A	C	A	ACD	BC
IC50 μg/ml	2C06 IgG	2.92	33.6	>100	>100	>100	>100	>100	>100	>100	>100	>100	>100	>100	>100	>100	>100
	2C09 IgG	2.07	44.2	>100	>100	>100	>100	>100	>100	>100	>100	>100	>100	>100	>100	>100	>100
	N123-VRC34.01	0.017	0.116	0.254	0.082	0.072	0.155	0.121	0.214	0.173	0.062	1.11	0.050	>100	0.134	0.039	0.035
IC80 μg/ml	2C06 IgG	10.4	>100	>100	>100	>100	>100	>100	>100	>100	>100	>100	>100	>100	>100	>100	>100
	2C09 IgG	10.9	>100	>100	>100	>100	>100	>100	>100	>100	>100	>100	>100	>100	>100	>100	>100
	N123-VRC34.01	0.084	0.641	1.05	0.317	0.323	1.03	0.414	0.832	1.21	0.604	>100	0.194	>100	1.25	0.14	>100

C

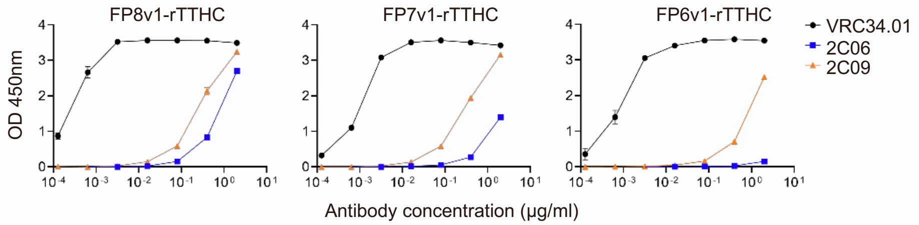

D Competition ELISA of 2C06 and 2C09 binding to BG505 DS-SOSIP with fusion peptide and base-binding antibodies

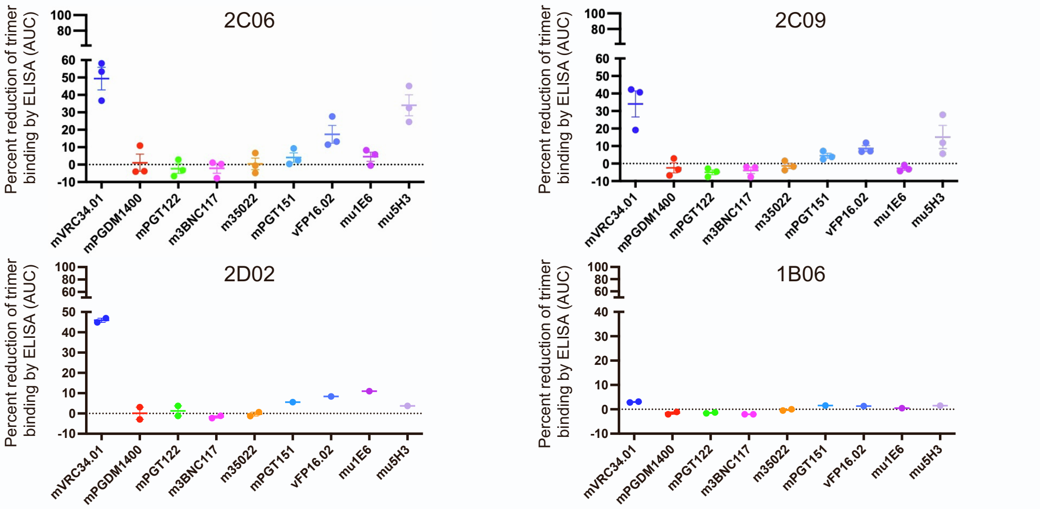

E Neutralization assessments against BG505.S241N as well as against all strains in 208-strain panel lacking a glycan at residue 241

	Viral strains	BG505.S241N	CNE56	929-28	T257-31	0077.v1.c16	3168.v4.c10	6471.v1.c16	25710-2.43
	Tier	2		2	2/3	2		3	1B/2
	Clade	A	AE	AG	AG	C	C	C	C
IC50 μg/ml	2C06	>100	>100	>100	>100	>100	>100	>100	>100
	2C09	>100	>100	>100	>100	>100	>100	>100	>100
	VRC34.01	0.758	>100	>100	>100	1.48	11.5	>100	0.366
IC80 μg/ml	2C06	>100	>100	>100	>100	>100	>100	>100	>100
	2C09	>100	>100	>100	>100	>100	>100	>100	>100
	VRC34.01	4.610	>100	>100	>100	>100	65.6	0>100	>100

Figure S2. Binding and neutralization data, related to Figures 1, 5, and 6.
(A) Neutralization assessments for 2D02 and 1B06 revealed no detectable neutralization activity.
(B) Neutralization assessments of VRC 018-elicited antibodies from donor N751 against a panel of viruses neutralized by most fusion peptide-directed antibodies.
(C) ELISA analysis of 2C06 and 2C09 IgG binding to fusion peptide. Measurements were carried out in triplicates, and mean and SE are reported for each data point.
(D) Competition ELISA revealed 2C06, 2C09, and 2D02 competed for binding to BG505 DS-SOSIP trimer with fusion peptide-directed antibodies and base binding antibody 1E6 or 5H3, whereas 1B06 did not compete with any antibody tested.
(E) Neutralization assessments focusing on strains in 208-strain panel lacking a glycan at residue 241.

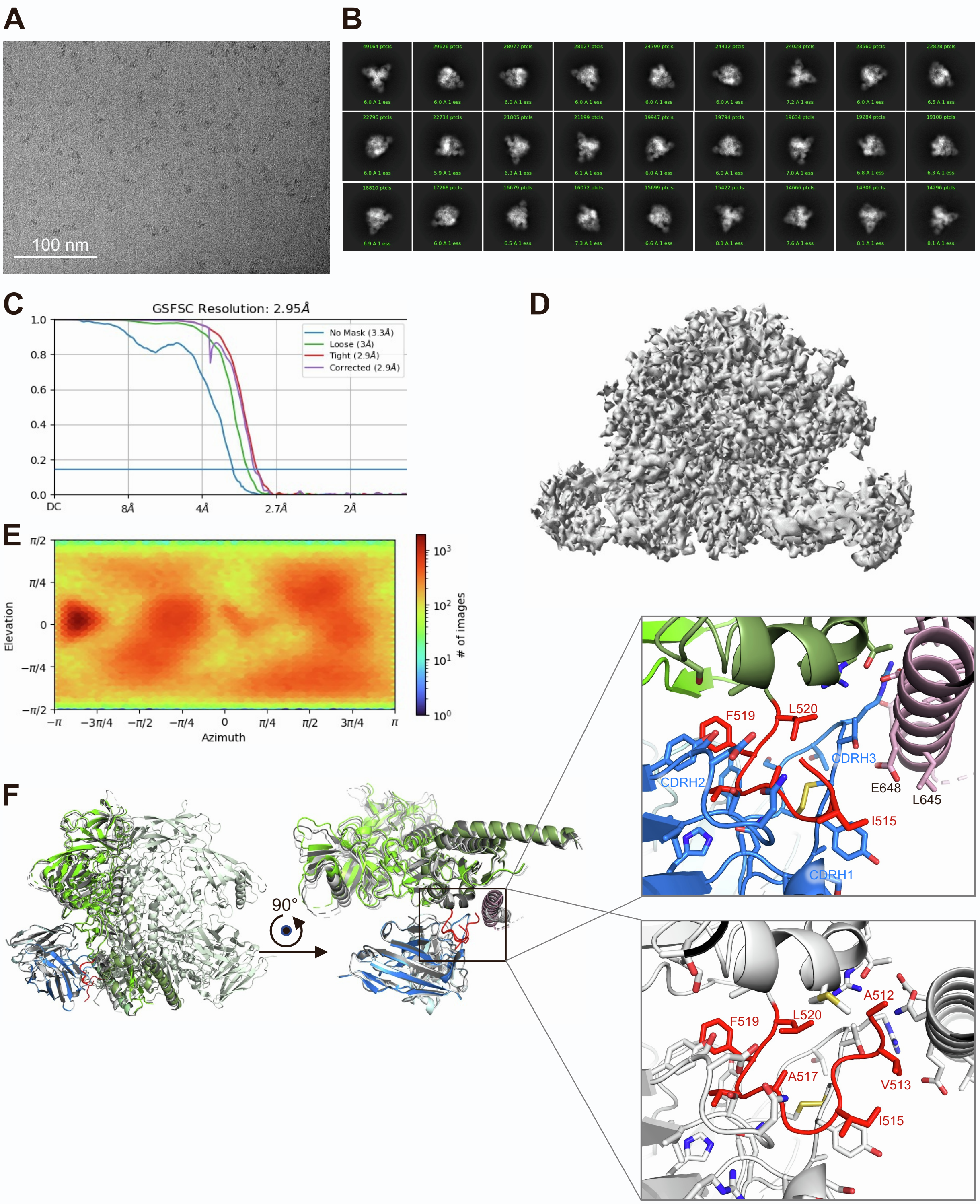

Figure S3. Cryo-EM validation for 2C06 in complex with BG505 DS-SOSIP, related to Figure 2.
(A) Representative micrograph. (B) 2D classes. (C) Fourier shell correlation (FSC) curves showing gold-standard FSC resolution at 2.95 Å. (D) 3D reconstruction density map at 2.95 Å from non-uniform refinement with C1 symmetry, showing antibody Fab binding near the trimer base at the fusion peptide-site of vulnerability. (E) Orientations of all particles used in the final refinement shown as a heatmap. (F) Superposition of three protomers revealing the trimer asymmetry induced by 2C06 binding. (Left) Protomers in right and back are superimposed on the one in left. (Middle) A 90° rotation of the left image to put the superimposed antibody chains at the bottom. Only the superimposed protomers are shown. One protomer is shown in colors, and the other two are in light or dark gray. (Right) Zoom-in views showing two slightly different modes of binding. The helix on the right is from a neighboring protomer.

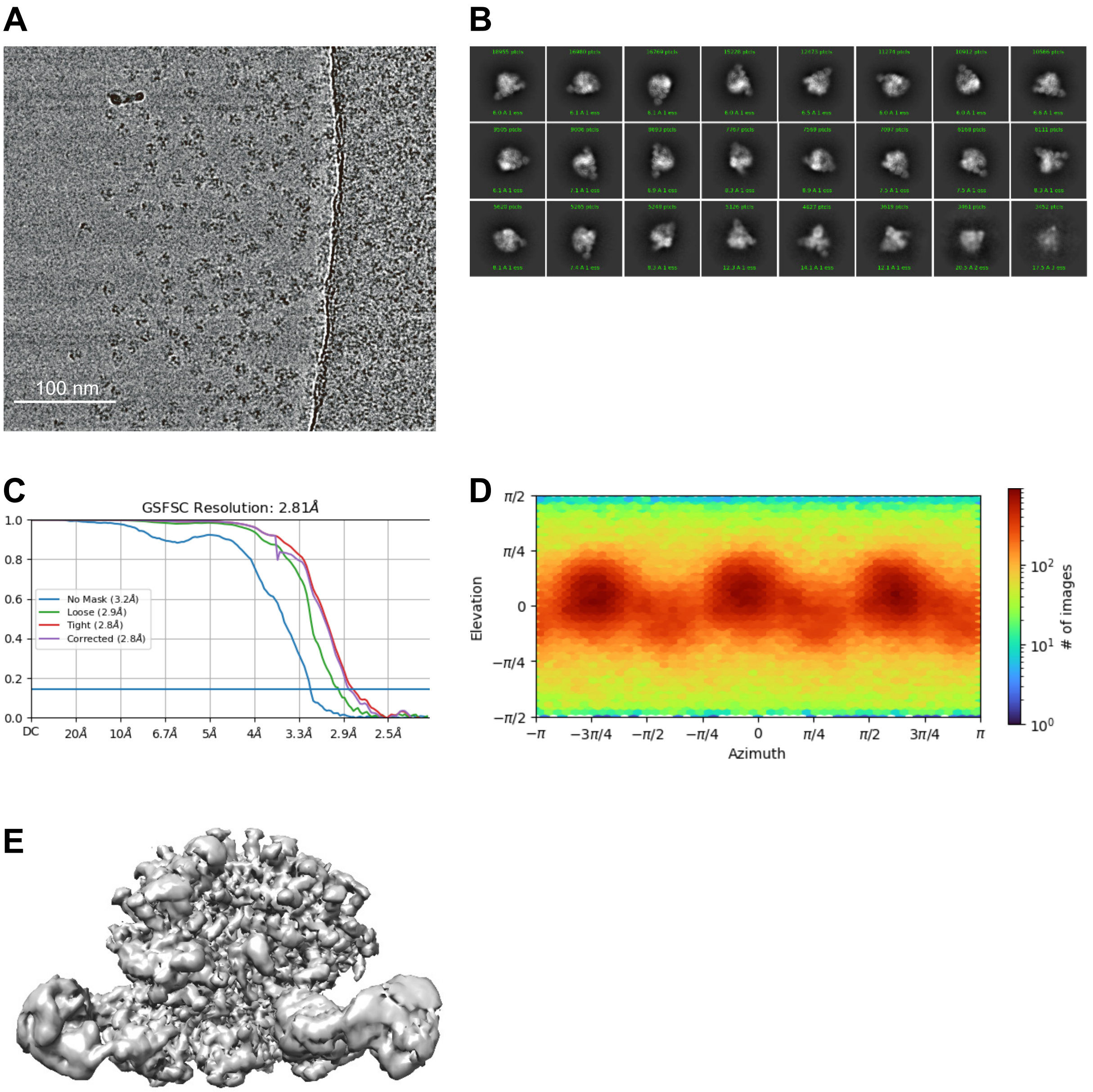

Figure S4. Cryo-EM validation for 2C09 in complex with BG505 DS-SOSIP, related to Figure 3.
(A) Representative micrograph. (B) 2D classes. (C) Fourier shell correlation (FSC) curves showing gold-standard FSC resolution at 2.81 Å. (D) Orientations of all particles used in the final refinement shown as a heatmap. (E) 3D reconstruction density map at 2.81 Å from non-uniform refinement with C3 symmetry, showing antibody Fab binding near the trimer base at the fusion peptide-site of vulnerability.

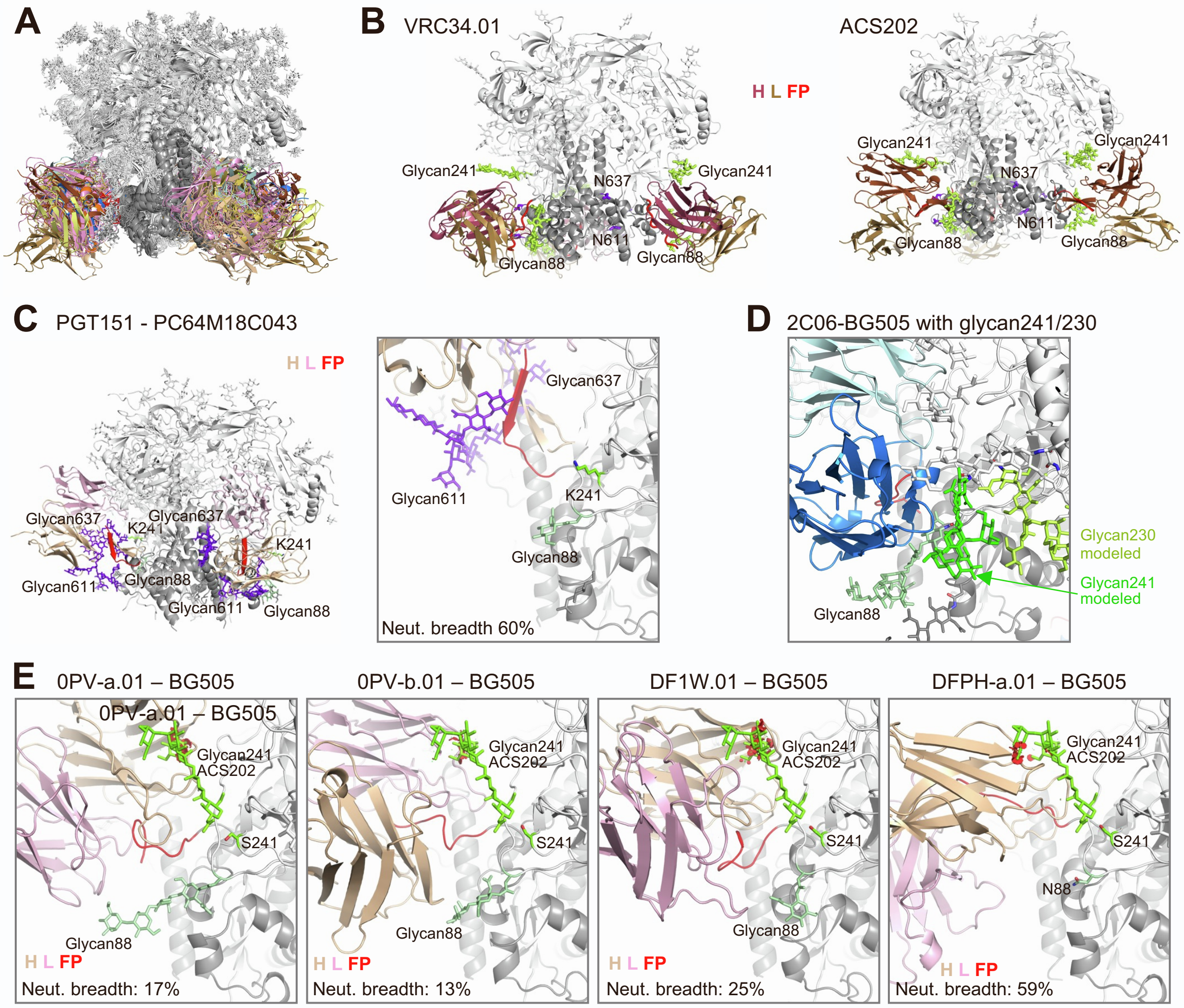

Figure S5. Fusion peptide-directed antibodies (except for PGT151) bind at a similar location and interact favorably with glycan88 but variably with glycan241, related to Figure 5.

(A) Structural superposition of fusion peptide-directed antibodies in complex with Env trimer. The structures, 2C06, 2C09, ACS202 (PDB: 6nc2), VRC34.01 (PDB: 6nc3), DFPH-a.01 (modeled based on DFPH-a.15 structure, PDB: 6n1w), DF1W-a.01 (PDB: 6mph), A12V163-a.01 (PDB: 6n1v), A12V163-b.01 (PDB: 6mpg), 0PV-a.01 (PDB: 6osy), 0PV-b.01 (PDB: 6ot1), 0PV-c.01 (PDB: 6nf2), and vFP16.02 (PDB: 6cdi), were aligned by one of the gp120 subunits (right).

(B) Binding of VRC34.01 and ACS202 involves both glycan88 and glycan241 (green sticks), but not glycan611 or glycan637, both not modeled in the structure, likely disordered. VRC34.01 and ACS202 bind in a similar location but with different orientations.

(C) PGT151 (PDB: 6dcq) binds to glycan611 and glycan637 (blue-purple sticks), away from glycan88 (green sticks) and the side chain of K241 (green sticks). Neutralization breadths were for IC50 <50 μg/ml in the 208-strain panel.

(D) Structure of 2C06-BG505 complex with glycans at 241 and 230, as well as all other potential glycans modeled as mannose 5. With adjustment of glycan conformations, both glycans at 241 and 230 could be modeled without clashing with each other or with the antibody.

(E) Vaccine-elicited NHP or murine antibodies all bind at a similar location to that of VRC34.01 and ACS202. The aligned glycan241 from the ACS202 complex (green sticks) has some minor clashes with antibodies 0PV-a.01, 0PV-b.01, DF1W.01, and DFPH-a.01, shown as red disks in Pymol (https://pymol.org/2/). These clashes are from the terminal mannose residues and can be avoided by minor shifts of the glycan, unlike those observed in 2C06 and 2C09 and other vaccine-elicited antibodies shown in Figure 5.

A

Position	Entropy	Most freq aa	BG505 aa	2C06 Contact atoms[b]	2C06 BSA[c]	2C09 Contact atoms	2C09 BSA	A12V163-a.01 Contact atoms	A12V163-a.01 BSA	A12V163-b.01 Contact atoms	A12V163-b.01 BSA	VRC34.01[d] Contact atoms	VRC34.01[d] BSA	vFP16.02 Contact atoms	vFP16.02 BSA	0PV-c.01 Contact atoms	0PV-c.01 BSA
512	0.082	Ala (96.5)[a]	Ala	C, O	4.78	C,O	11.9	N, CA, C, O, CB	158.6	CA, C, O	20.1	N(H), CA, C, O, CB	127.3	N, CA, C, O(H), CB	161.6	N(H,S), CA, C, O, CB	83.1
513	0.275	Val (80.6)	Val	N, CA, C, O, CB, CG2	26.9	CA, C, O, CB, CG1, CG2	87.6	N, CA, C, O, CB, CG1, CG2	86.2	N, CA, C, O, CB	22.9	N(H), CA, C, O(H), CB, CG1, CG2	114.5	N(H), CA, C, O(H), CB, CG1, CG2	106.9	N(H), CA, C, O, CB, CG1, CG2	97.4
514	0.226	Gly (84.9)	Gly	N, CA, C, O	19.9	N, CA	11.9	N(H), CA, C, O(H)	73.7	N, CA, C, O	46.4	N, CA, C, O	22.6	N(H), CA, C, O	42.6	N, CA, C, O(H)	90.7
515	0.411	Ile (50.0)	Ile	N, CA, C, O, CB, CG1, CG2, CD1	75.6	N(H), C, O(H), CB, CG1, CG2, CD1	30.7	N, CA, C, O, CB, CG1, CG2, CD1	154.6	N, CA, C, O(H), CB, CG1, CG2, CD1	125.4	N(H), CA, C, O(H), CB, CG1, CG2, CD1	153.4	N, CA, C, O, CB, CG1, CG2, CD1	140.6	N, CA, C, O, CB, CG1, CG2, CD1	134.5
516	0.023	Gly (99.0)	Gly	N, CA, C, O(H)	81.5	N(H), CA, C, O(H)	55.9	N, CA, C, O	25.1	N, CA, C, O	41.3	N, CA, C, O(H), CB, CG1, CG2	38.7	N, CA, C, O	38.2	N(H), CA, C, O(H)	67.1
517	0.043	Ala (98.1)	Ala	N, CA, C, O, CB	31.1	N, CA, C, O, CB	36.3	N, CA, C, O, CB	44.8	N(H), CA, C, O, CB	108.9	N(H), CA, C, O(H), CB	46.9	N, CA, C, O, CB	40.3	N(H), CA, C, O(H), CB	70.3
518	0.429	Val (45.1)	Val	N, CA, C, O(H), CB, CG1, CG2	146.2	N, CA, O, CB, CG1, CG2	151.4	CA, C, CB, CG1, CG2	74.6	N, CA, C, O, CB, CG1, CG2	122.9	N, CA, C, O, CB, CG1, CG2	91	N, CA, CB, CG1, CG2	34	N(H), CA, C, O, CB, CG1, CG2	145.4
519	0.183	Phe (85.9)	Phe	N, CA, C, O, CB, CG, CD1, CD2, CE1, CE2, CZ	106.4	N, CA, O, CB, CG, CD1, CD2, CE1, CE2, CZ	108.6	N, O, CG, CD1, CE1, CE2, CZ	67.9	N, CA, C, O, CB, CG, CD1, CD2, CE1, CE2, CZ	132.3	N(H), C, O(H), CB, CG, CD1, CD2, CE2	72.1	CB, CG, CD1, CD2, CE1, CE2, CZ	46.2	N(H), CA, C, O(H), CB, CD1, CD2, CE1, CE2, CZ	83.2
520	0.144	Leu (90.8)	Leu	N, CA, O, CB, CG, CD1, CD2	45.5	N, O, CB, CD1	65	CA, C, CB, CG, CD1, CD2	63.6	N	6.02	N, CA, O(H), CB, CD1	47.7	CD1	17.2	N, CA, O, CB, CG, CD1, CD2	62.4
535	0.472	Ile (47.6)	Met (17.6)	CG	5.52	SD, CE	17.7							CG	1.67	SD, CE	26.7
85	0.733	Val (49.8)	His (2.5)	N, CA, O, CB, CG, CD2, ND1, CE1, NE2	67.9	N, CA, CB, CG, CD2, ND1, CE1, NE2	62.1	N, CA, C, O, CB, CG, CD2, ND1, CE1, NE2	94.6	N, CA, C, O, CB, CG, CD2, ND1, CE1, NE2	74.4	N, CB, CG1, CG2	40.9	N, CB, CG, CD2, ND1, CE1, NE2	43.2	N(H), O, CB, CG, CD2, ND1, CE1, NE2	69
87	0.573	Glu (52.7)	Glu	CA, CB, CG, CD, OE1, OE2	77.3	CB, CG, CD, OE1, OE2(H,S)	85.6	N, CA, C, O, CB, CG, CD	46.2	N, CA, CB, CG, CD, OE1, OE2	77.3			CB, CG, CD, OE1, OE2	39.2	CG, OE1, OE2	46.7
640	0.743	Gly (24.8)	Gln (6.8)							CB, CG, CD, OE1, NE2	61.2						
644	0.729	Thr (26.5)	Gly (1.7)	CA, C, O	19.2												
648	0.547	Glu (54.9)	Glu	N, CA, CB, CG, CD, OE1	23.2												
651	0.417	Asn (69.2)	Asn	CB, CG, ND2	28.8												

a – Numbers in parentheses are the percentage frequency of the amino acid in the 2020 HIV-1 group M sequences.
b – Hydrogen bonds or salt bridges involved with the atom are designated as H or S, respectively.
c – BSA, buried surface area in $Å^2$ per residue calculated with PISA. 0 [color scale] 160 $Å^2$
d – VRC34.01 in complex with AMC011 Env, which has a Val at position 85 instead of His.

B His85 interactions with 2C09

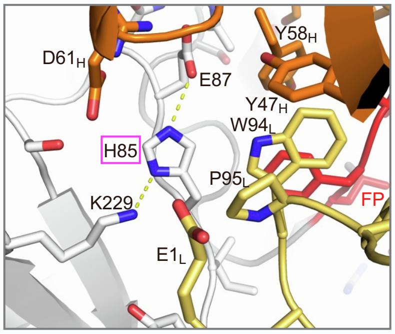

C His85 interactions with 0PV-c.01

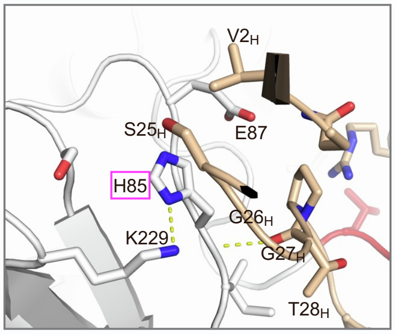

D

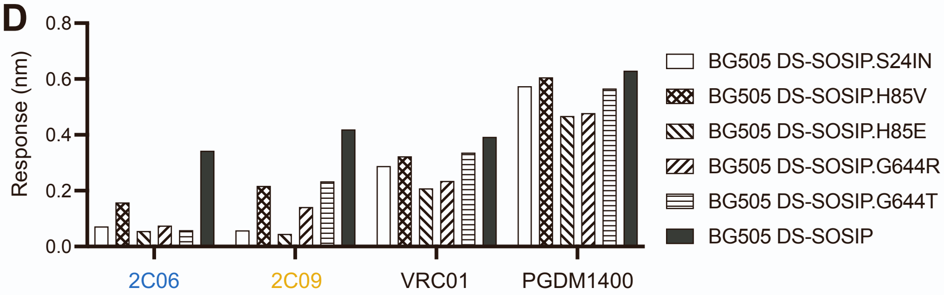

Figure S6. Analysis of entropy and binding interactions of the epitope residues for 2C06 and 2C09 in comparison with other fusion peptide-directed antibodies, related to Figure 6.
(A) Shannon entropy and contact surface areas of epitope residues for strain-specific and broadly neutralizing fusion peptide-directed antibodies. Residues analyzed include fusion peptide residues 512 to 520 and other residues involved in binding of 2C06 and 2C09 with normalized entropy above 0.4. Entropy values were calculated with Shannon Entropy-One (https://www.hiv.lanl.gov/content/sequence/ENTROPY/entropy_one.html) on the curated alignment of year 2020 HIV-1 M group Env of 5255 sequences (https://www.hiv.lanl.gov/content/sequence/NEWALIGN/align.html), and were normalized to have values between 0 and 1. Contact atoms and BSA were calculated with PISA (https://www.ebi.ac.uk/msd-srv/prot_int/cgi-bin/piserver). (B) His85 has tight interactions with 2C09 heavy and light chain residues, similar to that in the 2C06 complex. (C) In 0PV-c.01, most interactions with H85 side chain are from heavy chain residues S25, G26, and G27. There is space and flexibility to accommodate other amino acids at position 85, despite large BSA with H85. (D) BLI assays for antibody binding to mutants of BG505 DS-SOSIP, S241N, H85V, H85E, G644R, and G644T.

Table S1. Cryo-EM data and refinement statistics, related to Figures 2 and 3.

	2C06	2C09
**EMDB ID**	EMD-29725	EMD-29731
**PDB ID**	8G4M	8G4T
Data Collection		
Microscope	FEI Titan Krios	FEI Titan Krios
Voltage (kV)	300	300
Electron dose ($e^-/Å^2$)	58.06	58
Detector	Gatan K3 BioQuantum	Gatan K2 Summit
Pixel Size (Å)	0.83	1.11
Defocus Range (μm)	-0.8/-2.0	-1.0/-2.5
Magnification	105,000	29,000
Reconstruction		
Software	cryoSPARC v3.3.1	cryoSPARC v3.3.1
Particles	645,442	445,733
Symmetry	C1	C3
Resolution (Å) ($FSC_{0.143}$)	2.95	2.81
Refinement		
Software	Phenix 1.20	Phenix 1.20
Protein residues	2432	2437
Ligands	BMA: 7; NAG: 98; MAN: 14	BMA: 9; NAG: 105; MAN: 15
CC (box)	0.80	0.83
CC (mask)	0.88	0.88
R.m.s. deviations		
Bond lengths (Å)	0.003	0.004
Bond angles (°)	0.459	0.453
Validation		
Molprobity score	1.55	1.51
Clash score	4.25	4.00
Rotamer outliers (%)	2.43	1.38
Ramachandran		
Favored regions (%)	97.78	96.62
Allowed regions (%)	2.22	3.38
Disallowed regions (%)	0	0

Table S2. Epitope analysis for fusion peptide-directed antibodies in comparison to non-fusion peptide antibodies, related to Figure 6.

Epitope entropy was calculated as BSA weighted average of normalized Shannon's entropy based on 208-virus panel for the epitope residues of each antibody. Percentage of epitope overlap was calculated as number of overlapping epitope residues divided by the number of epitope residues for the antibody with the least number of epitope residues of the two. Epitope residue was defined as residue with BSA>0. Fusion peptide-directed antibodies have substantial epitope overlap, and they have similar epitope entropies, except PGT151, which binds at a slightly different location and has a lower epitope entropy. Non-fusion peptide antibodies have variable epitope entropies that correlates with neutralization breadth.

Category	PDB ID	Antibody	Epitope entropy	Neut breadth	Epitope BSA (Å^2)	Epitope overlap (%) with	
						2C06	2C09
Fusion peptide		2C06	0.29	<1%	1268	100	90
		2C09	0.26	<1%	1044	90	100
	6mpg	A12V163-b.01	0.28	0%	1083	84	87
	6n1v	A12V163-a.01	0.28	3%	1055	84	92
	6osy	0PV-a.01	0.26	17%	1108	71	71
	6ot1	0PV-b.01	0.26	13%	1017	67	57
	6nf2	0PV-c.01	0.27	23%	1320	62	71
	6mph	DF1W-a.01	0.26	25%	1068	75	65
	6nc2	ACS202	0.30	26%	902	62	74
	6cdi	vFP16.02	0.24	31%	1107	69	81
	6nc3	VRC34.01	0.27	50%	774	71	75
	6n1w	DFPH-a.1	0.27	59%	1179	88	69
	5fuu	PGT151	0.15	60%	1465	54	55
V1V2	3u2s	PG9	0.38	81%			
	5v8l	PGT145	0.31	75%			
	5vgj	VRC38.01	0.46	29%			
Glycan-V3	5aco	PGT128	0.38	69%			
	5cez	PGT121	0.34	65%			
	4jm2	PGT135	0.47	34%			
	6ozc	2G12	0.40	25%			
CD4-binding site	5fyj	VRC01	0.25	90%			
	4ydj	VRC13.01	0.16	83%			
	4ydk	VRC16.01	0.18	55%			
	5f9o	8ANC131	0.20	53%			
	4jan	CH103	0.23	53%			
	5t3z	IOMA	0.23	50%			
	5vn8	b12	0.29	43%			
	4ye4	HJ16	0.51	31%			
Silent face	6bf4	VRC-PG05	0.60	28%			
Subunit interface	5cez	35O22	0.29	45%			
	5cjx	8ANC195	0.21	67%			

Table S3. Analysis of antibody-trimer interfaces, by CDRs, related to Figures 2 and 3. Subunit interface buried surface areas (Å^2) were analyzed using PISA. Blank entries are 0, i.e. no binding interactions.

		Protomer 1		Protomer 2		Protomer 3		
	Chain	A gp41	C gp120	B gp41	G gp120	F gp41	I gp120	Subtotal
**2C06**	CDR H1	54.67						54.67
Antibody 1	CDR H2	164.67	129.49					294.16
(chains DE)	CDR H3	316.52	14.32			126.34		457.18
	L N-term		27.07					27.07
	CDR L1		87.45					87.45
	CDR L3	58.34	176.47					234.81
	FR L3		8.97					8.97
	DE total	594.2	443.77			126.34		1164.31
Antibody 2	CDR H1			72.27				72.27
(chains JK)	CDR H2			184.65	118.97			303.62
	CDR H3	204.07		280.25	13.11			497.43
	L N-term				7.71			7.71
	CDR L1				95.57			95.57
	CDR L3			59.48	176.94			236.42
	FR L3				7.66			7.66
	JK total	204.07		596.65	419.96			1220.68
Antibody 3	CDR H1					65.28		65.28
(chains HL)	CDR H2					198.22	122.92	321.14
	CDR H3			202		255.54	15.73	473.27
	L N-term						11.5	11.5
	CDR L1						92.69	92.69
	CDR L3					58.82	178.01	236.83
	FR L3						6.7	6.7
	HL total			202		577.86	427.55	1207.41
**2C09**	CDR H1	93.24						93.24
Antibody 1	CDR H2	164.88	98.03					262.91
(chains DE)	CDR H3	256.08	14.32					270.4
	L N-term		27.07					27.07
	CDR L1		83.25					83.25
	CDR L3	92.08	170.84					262.92
	FR L3		8.97					8.97
	DE total	606.28	402.48					1008.76
antibody	CDR H1			92.27				92.27
(chains JK)	CDR H2			164.61	100.5			265.11
	CDR H3			258.57	13.11			271.68
	L N-term				7.71			7.71
	CDR L1				83.33			83.33
	CDR L3			88.73	170.05			258.78
	FR L3				7.66			7.66
	JK total			604.18	382.36			986.54
Antibody	CDR H1					91.49		91.49
(chains HL)	CDR H2					166.05	98.03	264.08
	CDR H3					259.94	15.73	275.67
	L N-term						11.5	11.5
	CDR L1						83.74	83.74
	CDR L3					86.97	170.6	257.57
	FR L3						6.7	6.7
	HL total					604.45	386.3	990.75

Table S4. Analysis of antibody-trimer interfaces, by trimer sequence segments, related to Figures 2 and 3. Subunit interface buried surface areas (Å^2) were analyzed using PISA. Blank entries are 0, i.e. no binding interactions.

		Epitope subregions	Antibody 1 DE Heavy	Antibody 1 DE Light	Antibody 2 JK Heavy	Antibody 2 JK Light	Antibody 3 HL Heavy	Antibody 3 HL Light
**2C06**								
Protomer 1	A gp41	FP 512-520	469.77	64.41				
		524-542	109.63					
		592-648			149.14			
	C gp120	79-88	125.14	276.3				
		glycan88	249.6					
		227+229		45.91				
		241	19.63					
Protomer 2	B gp41	FP 512-520			489.37	67.96		
		524-542			154.52			
		588-648					142.4	
	G gp120	79-88			119.21	292.67		
		glycan88			247.2			
		227+229				19.07		
		241			20.68			
Protomer 3	F gp41	FP 512-520					455.95	67.28
		524-542					146.62	
		644-651	99.28					
	I gp120	79-88					125.64	299.58
		glycan88					246	
		227+229						20.52
		241					22.27	
	Subtotal		1073.05	386.62	1180.1	379.7	1138.9	387.38
**2C09**								
Protomer 1	A gp41	FP 512-520	463.99	91.9				
		524-542	70.96					
		592-648						
	C gp120	85-88	125.14	279.91				
		glycan88	285					
		229		10				
		241	19.63					
Protomer 2	B gp41	FP 512-520			470.71	90.22		
		524-542			71.48			
		588-648						
	G gp120	85-88			101.4	279.55		
		glycan88			285			
		229				7.5		
		241			15.99			
Protomer 3	F gp41	FP 512-520					469.98	89.36
		524-542					71.83	
		644-651						
	I gp120	85-88					102.66	276.51
		glycan88					285	
		229					0.17	8.55
		241					14.1	
	Subtotal		964.72	381.81	944.58	377.27	943.74	374.42

Table S5. Frequency of N-glycosylation sites in HIV-1 Env sequences, related to Figure 5. HIV-1 Env protein sequence alignment was from HIV sequence database (https://www.hiv.lanl.gov/content/sequence/NEWALIGN/ align.html) M group without recombinants, year 2020, a total of 5255 sequences. The aligned sequences were input to N-GlycoSite (https://www.hiv.lanl.gov/content/sequence/GLYCOSITE/glycosite.html), excluding NP[ST] and first position N in NN[ST][ST], with HXB2 as reference sequence.

Position in sequence alignment	HXB2 sequence	HXB2 residue number	Number of sequences with N-glycan sequon	Frequency of N-glycosite
10	h	9	19	0.36
13	w	11	1	0.02
14	r	12	1	0.02
41	i	27	19	0.36
43	s	29	185	3.52
44	a	30	1	0.02
45	t	31	7	0.13
46	-	31	1	0.02
47	-	31	1	0.02
48	-	31	1	0.02
52	-	31	3	0.06
54	-	31	2	0.04
55	-	31	1	0.02
56	-	31	1	0.02
58	e	32	16	0.30
74	e	47	18	0.34
76	t	49	619	11.78
82	c	54	1	0.02
87	k	59	1	0.02
96	n	67	1	0.02
126	v	85	15	0.29
128	v	87	1	0.02
131	-	87	1	0.02
132	N	88	5186	98.69
136	n	92	23	0.44
155	k	97	20	0.38
156	n	98	2	0.04
173	d	113	5	0.10
180	c	119	1	0.02
184	-	122	1	0.02
188	-	125	1	0.02
191	s	128	7	0.13
192	l	129	1	0.02
193	k	130	3353	63.81
195	t	132	55	1.05
196	d	133	872	16.59
197	l	134	167	3.18
198	k	135	1243	23.65
199	N	136	881	16.76
200	d	137	1268	24.13
201	t	138	824	15.68
202	n	139	760	14.46
203	t	140	812	15.45
204	N	141	778	14.80
205	s	142	743	14.14
206	-	142	466	8.87
207	-	142	312	5.94
208	-	142	250	4.76
209	-	142	160	3.04
210	-	142	135	2.57
211	-	142	111	2.11
212	-	142	71	1.35
213	-	142	63	1.20
215	-	142	12	0.23
217	-	142	6	0.11
218	-	142	6	0.11
219	-	142	13	0.25
220	-	142	6	0.11
221	-	142	15	0.29
222	-	142	2	0.04
223	-	142	3	0.06
224	-	142	2	0.04
225	-	142	4	0.08
226	-	142	3	0.06
227	-	142	4	0.08
228	-	142	4	0.08
231	-	142	2	0.04
232	-	142	1	0.02
233	-	142	1	0.02
236	-	142	1	0.02
238	-	142	2	0.04
239	-	142	5	0.10
240	-	142	9	0.17
241	-	142	21	0.40
242	-	142	38	0.72
243	-	142	31	0.59
244	-	142	54	1.03
245	-	142	52	0.99
246	-	142	91	1.73
247	-	142	124	2.36
248	-	142	132	2.51
249	-	142	203	3.86
250	-	142	86	1.64
251	s	143	593	11.28
252	s	144	515	9.80
253	g	145	590	11.23
254	r	146	237	4.51
255	m	147	473	9.00
256	i	148	203	3.86
257	m	149	268	5.10
258	e	150	80	1.52
259	k	151	15	0.29
260	g	152	2	0.04
261	e	153	1	0.02

Position in sequence alignment	HXB2 sequence	HXB2 residue number	Number of sequences with N-glycan sequon	Frequency of N-glycosite
268	N	156	5030	95.72
272	N	160	4741	90.22
273	i	161	3	0.06
274	s	162	1	0.02
276	s	164	10	0.19
277	i	165	1	0.02
281	-	166	1	0.02
288	g	167	1	0.02
289	k	168	1	0.02
291	q	170	1	0.02
292	k	171	10	0.19
293	e	172	1	0.02
295	y	173	1	0.02
299	f	176	1	0.02
300	y	177	1	0.02
303	l	179	1	0.02
307	p	183	1	0.02
308	i	184	1	0.02
309	d	185	293	5.58
310	N	186	723	13.76
311	-	186	199	3.79
312	-	186	287	5.46
313	-	186	184	3.50
314	-	186	95	1.81
315	-	186	34	0.65
316	-	186	12	0.23
317	-	186	8	0.15
318	-	186	12	0.23
319	-	186	10	0.19
320	-	186	9	0.17
322	-	186	3	0.06
323	-	186	6	0.11
324	-	186	1	0.02
325	-	186	3	0.06
326	-	186	1	0.02
328	-	186	1	0.02
329	-	186	1	0.02
330	-	186	3	0.06
331	-	186	1	0.02
332	-	186	2	0.04
334	-	186	3	0.06
335	-	186	3	0.06
336	-	186	4	0.08
337	-	186	8	0.15
338	-	186	9	0.17
339	-	186	16	0.30
340	-	186	26	0.49
341	-	186	44	0.84
342	-	186	104	1.98
343	-	186	218	4.15
344	-	186	301	5.73
345	-	186	453	8.62
346	-	186	511	9.72
347	d	187	1505	28.64
348	t	188	1490	28.35
349	t	189	8	0.15
350	s	190	16	0.30
353	l	193	1	0.02
355	s	195	7	0.13
356	c	196	4	0.08
357	N	197	5145	97.91
358	t	198	1	0.02
374	k	207	2	0.04
395	k	227	1	0.02
397	n	229	59	1.12
401	-	229	1	0.02
402	N	230	2153	40.97
404	t	232	86	1.64
407	N	234	4098	77.98
411	t	236	10	0.19
413	p	238	7	0.13
416	N	241	5072	96.52
440	s	256	1	0.02
441	t	257	1	0.02
447	N	262	5220	99.33
449	s	264	4	0.08
452	e	267	1	0.02
453	e	268	41	0.78
457	-	268	1	0.02
464	e	269	28	0.53
467	-	269	1	0.02
475	N	276	5016	95.45
477	t	278	3	0.06
478	d	279	5	0.10
479	n	280	1	0.02
516	a	281	1	0.02
518	t	283	1	0.02
524	N	289	3671	69.86
529	-	289	4	0.08
531	s	291	6	0.11
533	e	293	230	4.38
535	N	295	3049	58.02
539	p	299	1	0.02
540	n	300	4	0.08

Position in sequence alignment	HXB2 sequence	HXB2 residue number	Number of sequences with N-glycan sequon	Frequency of N-glycosite
541	N	301	4969	94.56
542	n	302	74	1.41
544	r	304	1	0.02
568	g	321	2	0.04
569	k	322	1	0.02
588	h	330	8	0.15
591	N	332	4207	80.06
593	s	334	670	12.75
594	r	335	11	0.21
597	k	337	303	5.77
598	w	338	1	0.02
600	N	339	3615	68.79
601	n	340	10	0.19
602	t	341	4	0.08
604	k	343	47	0.89
605	q	344	138	2.63
606	i	345	1	0.02
607	a	346	7	0.13
609	k	348	1	0.02
611	r	350	6	0.11
612	-	350	2	0.04
617	e	351	6	0.11
618	q	352	1	0.02
620	g	354	378	7.19
621	-	354	14	0.27
622	-	354	5	0.10
623	-	354	6	0.11
624	-	354	1	0.02
628	-	354	3	0.06
630	-	354	4	0.08
631	-	354	7	0.13
632	-	354	8	0.15
633	n	355	211	4.02
634	N	356	3992	75.97
635	k	357	25	0.48
636	t	358	232	4.41
637	i	359	3	0.06
638	i	360	166	3.16
639	f	361	1	0.02
640	k	362	2177	41.43
642	-	362	3	0.06
643	q	363	304	5.78
653	t	373	2	0.04
655	h	374	1	0.02
656	s	375	1	0.02
662	e	381	1	0.02
668	N	386	4523	86.07
670	t	388	1	0.02
672	q	389	2	0.04
674	l	390	1	0.02
675	f	391	1	0.02
676	N	392	4100	78.02
677	s	393	89	1.69
678	t	394	216	4.11
679	w	395	94	1.79
680	f	396	1387	26.39
681	N	397	642	12.22
682	s	398	1722	32.77
683	t	399	381	7.25
684	w	400	382	7.27
685	s	401	518	9.86
686	t	402	1112	21.16
687	e	403	582	11.08
688	g	404	598	11.38
689	s	405	451	8.58
690	N	406	301	5.73
691	n	407	262	4.99
692	t	408	237	4.51
693	-	408	102	1.94
694	-	408	41	0.78
696	-	408	4	0.08
700	-	408	1	0.02
703	-	408	1	0.02
706	-	408	1	0.02
709	-	408	3	0.06
710	-	408	2	0.04
712	-	408	2	0.04
713	-	408	6	0.11
714	-	408	6	0.11
715	-	408	19	0.36
716	-	408	27	0.51
717	-	408	25	0.48
718	-	408	31	0.59
719	-	408	32	0.61
720	-	408	52	0.99
721	-	408	89	1.69
722	e	409	140	2.66
723	g	410	469	8.92
724	s	411	2247	42.76
725	d	412	48	0.91
726	t	413	1318	25.08
728	t	415	5	0.10
731	p	417	1	0.02
733	r	419	1	0.02

Position in sequence alignment	HXB2 sequence	HXB2 residue number	Number of sequences with N-glycan sequon	Frequency of N-glycosite
739	n	425	1	0.02
756	s	440	1	0.02
758	q	442	1524	29.00
759	i	443	1	0.02
760	r	444	462	8.79
762	s	446	370	7.04
764	N	448	4519	85.99
776	g	459	3	0.06
777	n	460	694	13.21
778	s	461	1101	20.95
779	n	462	1552	29.53
780	N	463	1044	19.87
781	-	463	189	3.60
782	-	463	102	1.94
783	-	463	47	0.89
784	-	463	2	0.04
788	-	463	7	0.13
804	-	463	1	0.02
805	-	463	1	0.02
806	-	463	5	0.10
808	-	463	11	0.21
809	-	463	8	0.15
810	-	463	13	0.25
811	-	463	16	0.30
812	-	463	13	0.25
813	-	463	23	0.44
814	-	463	43	0.82
815	-	463	51	0.97
816	-	463	50	0.95
817	-	463	127	2.42
818	-	463	1617	30.77
819	e	464	5	0.10
820	s	465	1101	20.95
833	n	478	4	0.08
843	v	488	1	0.02
855	t	499	1	0.02
952	q	567	1	0.02
984	g	597	1	0.02
994	a	607	7	0.13
998	N	611	5179	98.55
999	a	612	1	0.02
1000	s	613	36	0.69
1003	-	615	11	0.21
1004	-	615	15	0.29
1005	-	615	3	0.06
1006	-	615	1	0.02
1007	-	615	3	0.06
1009	-	615	1	0.02
1012	-	615	1	0.02
1013	-	615	2	0.04
1014	-	615	1	0.02
1015	-	615	19	0.36
1016	N	616	4780	90.96
1017	k	617	3	0.06
1018	s	618	287	5.46
1024	-	623	1	0.02
1025	-	623	1	0.02
1026	N	624	36	0.69
1027	h	625	5077	96.61
1038	i	635	1	0.02
1040	N	637	5082	96.71
1043	s	640	1	0.02
1048	s	644	1	0.02
1052	e	648	1	0.02
1083	n	656	1	0.02
1094	k	665	5	0.10
1101	n	671	1	0.02
1104	N	674	687	13.07
1130	v	698	1	0.02
1142	n	706	5	0.10
1157	-	718	5	0.10
1188	r	740	1	0.02
1189	d	741	6	0.11
1190	r	742	4	0.08
1191	d	743	245	4.66
1192	r	744	3	0.06
1195	i	746	6	0.11
1199	N	750	22	0.42
1212	s	762	16	0.30
1215	l	765	1	0.02
1223	d	773	4	0.08
1227	i	777	1	0.02
1246	r	787	2	0.04
1274	n	798	35	0.67
1285	k	808	5	0.10
1286	n	809	11	0.21
1292	l	815	1	0.02
1293	N	816	2149	40.89
1295	t	818	1	0.02
1297	i	820	1	0.02
1301	e	824	159	3.03
1312	q	834	1	0.02
1332	h	842	3	0.06
